# A feasibility randomized controlled trial of an individually delivered, peer support intervention to reduce the impact of psychosis stigma and discrimination for people with psychosis: the let's talk study

**DOI:** 10.1017/S0033291724002605

**Published:** 2024-12

**Authors:** Melissa Pyle, Patrick W. Corrigan, Lisa Wood, Stephen Pilling, Elizabeth Murphy, Gillian Macafee, Kate Kelly, Rory Byrne, Eleanor Dunbar, Emily Jones, Jemma Hudson, Wendy Jones, Raj Hazzard, Jon E. Larson, Graeme MacLennan, James Swingler, Sarah Peters, Anthony P. Morrison

**Affiliations:** 1 The Psychosis Research Unit, Department of Psychology, Greater Manchester Mental Health NHS Foundation Trust, Prestwich, M25 3BL, UK; 2 Division of Psychology and Mental Health, University of Manchester, Zochonis Building, Manchester, M13 9PL, UK; 3 Department of Psychology, Illinois Institute of Technology, 10 West 35th Street, Chicago, IL 60616, USA; 4 Division of Psychiatry, University College London, 149 Tottenham Court Road, London, W1T 7NF, UK; 5 Research and Development, Northeast London NHS Foundation Trust, Goodmayes Hospital, Barley Lane, Ilford, Essex, IG3 8XJ, UK; 6 Headspace Bolton C.I.C, 27 Bradshawgate, Bolton, BL11EL, UK; 7 The Centre for Healthcare Randomised Trials, Health Services Research Unit, University of Aberdeen, Aberdeen, UK; 8 McPin Foundation, 7-14 Great Dover Street, London, SE1 4YR, UK

**Keywords:** peer support, psychosis, randomized controlled trial, stigma intervention, stigma

## Abstract

**Background:**

Stigma of mental health conditions hinders recovery and well-being. The Honest, Open, Proud (HOP) program shows promise in reducing stigma but there is uncertainty about the feasibility of a randomized trial to evaluate a peer-delivered, individual adaptation of HOP for psychosis (Let's Talk).

**Methods:**

A multi-site, Prospective Randomized Open Blinded Evaluation (PROBE) design, feasibility randomised controlled trial (RCT) comparing the peer-delivered intervention (Let's Talk) to treatment as usual (TAU). Follow-up was 2.5 and 6 months. Randomization was via a web-based system, with permuted blocks of random size. Up to 10 sessions of the intervention over 10 weeks were offered. The primary outcome was feasibility data (recruitment, retention, intervention attendance). Primary outcomes were analyzed by intention to treat. Safety outcomes were reported by as treated status. The study was prospectively registered: https://doi.org/10.1186/ISRCTN17197043.

**Results:**

149 patients were referred to the study and 70 were recruited. 35 were randomly assigned to intervention + TAU and 35 to TAU. Recruitment was 93% of the target sample size. Retention rate was high (81% at 2.5 months primary endpoint), and intervention attendance rate was high (83%). 21% of 33 patients in Let's talk + TAU had an adverse event and 16% of 37 patients in TAU. One serious adverse event (pre-randomization) was partially related and expected.

**Conclusions:**

This is the first trial to show that it is feasible and safe to conduct a RCT of HOP adapted for people with psychosis and individual delivery. An adequately powered trial is required to provide robust evidence.

## Introduction

Stigma is defined as a personal attribute that is deeply discrediting, resulting in a person or group being discounted (Goffman, 1963). Public stigma can personally impact people with lived experience (PWLE) of a mental health condition and there is a global call to end mental health stigma (Thornicroft et al., 2022; Thornicroft, Sunkel, & Milenova, 2024).

The term *personal stigma* encompasses three stigma experiences: perceived, experienced, and internalized stigma (IS) (Brohan, Slade, Clement, & Thornicroft, 2010). Perceived stigma is the perception of stigmatizing attitudes from others and the degree to which a PWLE believes others view them this way (LeBel, 2008). Experienced stigma refers to discrimination i.e., the direct unfair and unjust treatment of another person across one or many life domains (Thornicroft, Brohan, Rose, Sartorius, & Leese, 2009). IS is a personal reaction to public stigma where stigma becomes assimilated into self-identity (Corrigan & Watson, 2002). Internalization of stigmatized beliefs (e.g. incompetence) or emotions (e.g. shame) can erode self-esteem and result in PWLE questioning why they should try to achieve desired life goals (Corrigan, Larson, & Rusch, 2009). For people with psychosis, IS is associated with an increase in psychological difficulties including depression and suicidality, and a reduction in overall wellbeing including personal recovery (Eliasson, McNamee, Swanson, Lawrie, & Schwannauer, 2021).

A mental health condition is not immediately recognizable on meeting a person and disclosure decisions can be difficult. Secrecy, shame, and social withdrawal may become coping mechanisms and create disclosure dilemmas (Corrigan, Sokol, & Rusch, 2013; Vauth, Kleim, Wirtz, & Corrigan, 2007). Psychosis is one of the most stigmatized mental health conditions (Wood, Birtel, Alsawy, Pyle, & Morrison, 2014) and has been subject to pernicious stereotyping in the media (Bowen, Kinderman, & Cooke, 2019) making disclosure particularly challenging (Pyle & Morrison, 2013).

Three meta-analyses have shown promise for psychosocial interventions that are designed to reduce IS; however, the evidence base is in its infancy and requires methodically robust studies (Luo, Li, Yang, Chen, & Zhao, 2022; Tsang et al., 2016; Wood, Byrne, Varese, & Morrison, 2016). Many IS interventions have been designed to be delivered by professionals, but this may pathologise IS. A peer support worker (PSW), who has lived experience of a mental health condition, may be better placed to deliver stigma interventions as they are credible role models who can directly challenge the legitimacy of stereotypes within a mutual and non-hierarchical relationship (Pyle, Pilling, Machin, Allende-Cullen, & Morrison, 2018). A number of meta-analyses have indicated that peer support (PS) may be an effective approach to reducing the harmful effects of stigma, including reducing IS and disclosure distress (Burke, Pyle, Machin, Varese, & Morrison, 2019; Sun, Yin, Li, Liu, & Sun, 2022; White et al., 2020), and improving stigma related variables of recovery, empowerment (White et al., 2020), and self-efficacy (Burke et al., 2019).

One peer-led approach is The Honest Open, Proud (HOP) program, which is a group-based intervention that aims to aid mental health disclosure decision making. Whilst stigma can make disclosure decisions challenging (Rüsch & Kösters, 2021) a successful disclosure can increase access to supportive relationships and reduce social stigma (Corrigan & Matthews, 2003). HOP considers disclosure a personal decision that may change over time and a decision that should be made by carefully balancing potential benefits and costs of disclosure (Scior, Rüsch, White, & Corrigan, 2020). A meta-analysis of five HOP RCTs evaluated effects on three outcomes: stigma stress, which is the extent to which a person perceives stigma-related harm to be greater than their coping resources (Rüsch et al., 2009), IS and depression. Results showed a significant medium effect size for reduced stigma at end of treatment and a significant small effect size for reduced IS at follow-up (Rüsch & Kösters, 2021). Whilst HOP shows promise, the evidence base remains limited and some HOP trials have experienced recruitment challenges, which may be attributable to the group nature of the intervention (Rüsch & Kösters, 2021). Whilst group delivery of HOP is well established, a significant adaptation in delivery mode from a group to individual delivery should carefully consider the potential for any adverse effects not previously assessed or accounted for in HOP protocols. Psychological intervention trials have come under scrutiny regarding the accuracy and transparency of adverse events reporting (Duggan, Parry, McMurran, Davidson, & Dennis, 2014) and transparent details of adverse event definitions, identification methods and rates should be provided in psychosocial intervention trials.

The UK's National Institute for Care Excellence (NICE) Guideline CG178 recognizes the need to address stigma for people with psychosis and makes a specific research recommendation to evaluate the effectiveness of peer support interventions (National Institute for Health and Care Excellence, 2014). This study aims to investigate the feasibility of a randomized controlled trial (RCT) of HOP for people with psychosis, adapted for individual delivery by PSWs in the UKs National Health Service (NHS).

## Methods

### Study design

We did a multicenter parallel group, single-blind, two-armed, feasibility RCT recruiting individuals at two UK NHS Trusts (Greater Manchester and Northeast London) with 2.5 (end of treatment) and 6-month follow-up. This trial was approved by the South Central–Berkshire-B REC on 27 June 2021 (reference: 21/SC/0232). The study protocol was approved by an independent Trial Steering and Data Monitoring Committee (TS/DMC) and is available in the appendix (pp. 2–20).

### Participants

Eligible participants were aged 16+; help-seeking; met ICD-10 F20-F29 Schizophrenia spectrum diagnosis criteria, or were in receipt of Early Intervention in Psychosis services; under the care of mental health services; at least moderate levels of disclosure distress as operationalized as a score of >3 on a single disclosure distress scale (DDS) (Rüsch et al., 2014); and at least moderate levels of IS operationalized as a score of ⩾3 on at least one of the IS domains on the Semi-structured Interview Measure of Stigma (SIMS) (Wood, Burke, Byrne, Enache, & Morrison, 2016). All participants provided informed consent before their participation in the trial.

Exclusion criteria were primary diagnosis of alcohol or substance dependency; diagnosis of moderate to severe learning disability; diagnosis of organic psychosis; non-English speaking where this prevented providing informed consent or completing questionnaires validated in English; and immediate risk to self or others determined by the NHS care team. We did not exclude participants based on comorbid psychiatric diagnoses.

Participants were recruited from NHS mental health services providing care to people with experience of psychosis. A broad approach was taken to recruitment and where possible all potentially eligible service users within a service were offered the study. A broad definition was used to define a referral, which was classed as verbal permission from the service user for their basic contact and eligibility details to be shared with the research assistant (RA). The eligibility check on the DDS and SIMS was completed by the RA at the baseline assessment, which typically took place in the participant's home or via a remote method such as the telephone or videoconferencing.

Remote working practices were required in response to the covid-19 pandemic, including remotely delivered assessments or intervention for participants at elevated risk from covid-19. Participants were required at consent to specify their preferred mode of intervention delivery (in-person or remote). Participants allocated to the intervention arm received the mode specified at consent unless a covid-19 factor prevented this i.e. a participant contracting covid-19 and isolating.

### Randomization and masking

Participants were randomly allocated in a 1:1 ratio to receive the intervention plus treatment as usual (TAU), or TAU alone. Allocation was randomly assigned via a secure and web-based system developed by the Clinical Trials Unit (Centre for Healthcare Randomised Trials [CHaRT]; Aberdeen UK). Randomization was in permuted blocks of random size and was stratified by center and baseline preference for intervention delivery mode. Randomization was independent and concealed at the individual level and follow-up assessments were completed by RA's blind to the randomization outcome.

### Procedures

For this study, the name HOP was amended to ‘Let's Talk’ following consultation with PWLE. Participants allocated to the intervention were offered up to 10 sessions over a 10-week window, with the option of one booster session before the 6-month follow-up. Sessions were typically once a week. Participants were offered flexibility regarding assessment or intervention appointment date, time, and venue, this included the offer of a home visit where risk reasons did not prevent this. The intervention was manualized and structured around a workbook. The manual promoted a flexible and collaborative approach and whilst the progression through the workbook was linear, a participant could prioritize or return to a particular section of the workbook. Central to the manual were peer principles (Gillard et al., 2017). Full details of the intervention can be found in the appendix (appendix pp. 21–23). Following each session, PSWs self-assessed completion of workbook strategies and adherence to peer principles using the Principle Based Fidelity Index (Gillard et al., 2021). With consent, sessions were audio recorded and independently rated using an adapted version of the HOP fidelity scale (Corrigan et al., 2013; Rüsch et al., 2014) (appendix pp. 24–35). Supervision was provided to PSWs in group format on a weekly basis by a peer specialist and a clinical psychologist.

### Outcomes

Our primary outcomes were recruitment, retention to follow-up at the primary endpoint (2.5 months) for two proposed primary outcomes and intervention attendance. We applied three-stage progression criteria to determine feasibility; we agreed with the Trial Steering and Data Monitoring Committee our *a priori* criteria.

We collected baseline self-report demographic data. The method used to collect gender identity data was self-report. We did not collect assigned sex at birth as we determined this was not required for the interpretation of the study results. We specified two candidate primary outcomes for a definitive trial based on the HOP efficacy literature; these were total score on the SIMS (Wood et al., 2016) and stigma stress (Rüsch et al., 2009) at end of treatment (2.5 month follow-up). The SIMS has 11-items covering the following domains: perceived stigma (1 item), experienced stigma (1 item), and internalized stigma (IS; 8 items). The first item ‘understanding of stigma’ is not scored. The IS items cover the impact of perceived and experienced stigma on self-esteem, safety behaviors/avoidance, relationships, experiences of psychosis, treatment, positive impacts of stigma, and personal recovery. Items are scored between 0 (not present) to 4 (severe). The SIMS is a valid and reliable measure to assess change in stigma experienced by people with psychosis (Wood et al., 2016). Factor analyses revealed a one factor solution of total stigma (sum of all 10 items that are scored). For our study, we present the SIMS total and the perceived, experienced, and internalized stigma subtotals. The interview format confers the advantage of establishing a meaningful conversation about stigma and we consulted our service user reference group (SURG) regarding the choice of IS outcome and the SIMS was the preferred choice for this reason.

All secondary outcomes were collected at baseline, 2.5 month and 6-month follow-up. In addition to stigma stress and SIMS we collected: disclosure distress using a single item question (Rüsch et al., 2014), service user defined recovery (Process of Recovery Questionnaire [QPR] (Law, Neil, Dunn, & Morrison, 2014), depression (Calgary Depression Rating Scale (Addington, Addington, & Schissel, 1990), social interaction anxiety (Social Interaction Anxiety Scale [SIAS] (Mattick & Clarke, 1998), empowerment (Rogers Empowerment Scale [RES] (Rogers, Chamberlin, Ellison, & Crean, 1997)), quality of life (Manchester Short Assessment of Quality-of-Life [MANSA] (Priebe, Huxley, Knight, & Evans, 1999), service utilization using the economic patient questionnaire (Davies et al., 2008) and health status using the EQ5D-5L (Bobes, García-Portilla, Sáiz, Bascarán, & Bousoño, 2005). We also collected data on two potential mediators of internalized shame (Internalised Shame Scale [ISS] (Cook, 1987)) and the Self-Esteem Rating Scale [SERS] (Lecomte, Corbiere, & Laisne, 2006). A measure of psychosis symptoms was not included since this was not determined to be a potential outcome for a future trial. Our SURG have recommended that for a stigma intervention trial inclusion of a psychosis measure would be inconsistent with the intervention content and the aims of the study (Morrison et al., 2016).

Safety outcomes were adverse and serious adverse events by study team report and thoroughly screening each participants electronic patient records from point of consent to trial exit. We provide complete definitions of adverse events in the study protocol in the (appendix pp. 13–14). We collected acceptability data via qualitative interviews with participants and peer support workers and will report elsewhere.

### Statistical analysis

A sample size of 60 participants (30 per treatment arm) was determined to be sufficient to inform sample size estimates for a future trial (Browne, 1995) as well as the feasibility aims of the trial. To allow 20% attrition, we intended to randomize 75 participants. A priori progression criteria were agreed with our independent TS/DMC and funder as follows: recruitment of at least 80% of the planned population (green), 60–79% (amber), or less than 60% (red); retention of participants within the study with end of treatment data on the semi-structured interview measure of stigma and stigma stress with at least 80% (green), 60–79% (amber), or less than 60% (red); and at least 80% of receiving at least two sessions of the Let's Talk program (green), 60–79% (amber), or less than 60% (red).There was no formal power calculation to detect treatment differences, given the focus was not on hypothesis testing. The analysis followed a pre-specified statistical analysis plan approved by the committees/DMC, and published on the Clinical Trials Unit (CTU) here: https://www.abdn.ac.uk/hsru/what-we-do/trials-unit/statistical-analysis-plans-611.php.

Statistical analyses were based on intention-to-treat. Safety was analyzed based on treatment received (as treated), which was defined as receiving at least one session of the intervention. Progression criteria was summarized using descriptive statistics for the number of participant referrals, recruitment rate, retention of participants for SIMS and stigma stress, and number of Let's Talk sessions attended. Descriptive statistics are reported for safety data, number of participants receiving allocated interventions, frequency of delivery of intervention strategies, peer fidelity index, loss to follow-up, and hospital admissions. Session data is also reported by mode of delivery. Descriptive data from baseline and follow-up assessments were summarized as the mean (s.d.) or medians (IQR) for continuous data and frequencies and percentages for categorical variables. Outcomes were analyzed using repeated measures, mixed-effects regression models correcting for baseline score and time as categorical fixed effects with center and participant as a random effect. We used all available data with missing baseline data imputed with center-specific mean, and treatment effects were estimated at each time point with a treatment-by-time interaction. Results are presented as mean differences (MD) and 95% confidence intervals (CIs) and using standardized mean differences. All analyses were done in Stata (version 17) (StataCorp, 2019). The study was prospectively registered on 8 August 2021, on the ISRCTN registry, https://doi.org/10.1186/ISRCTN17197043.

### Role of funding source

The Let's Talk study was funded by the UK National Institute for Health Research (NIHR200460). The funder of the study had no role in study design, data collection, data analysis, data interpretation, or writing of the report.

## Results

**Figure 1.**
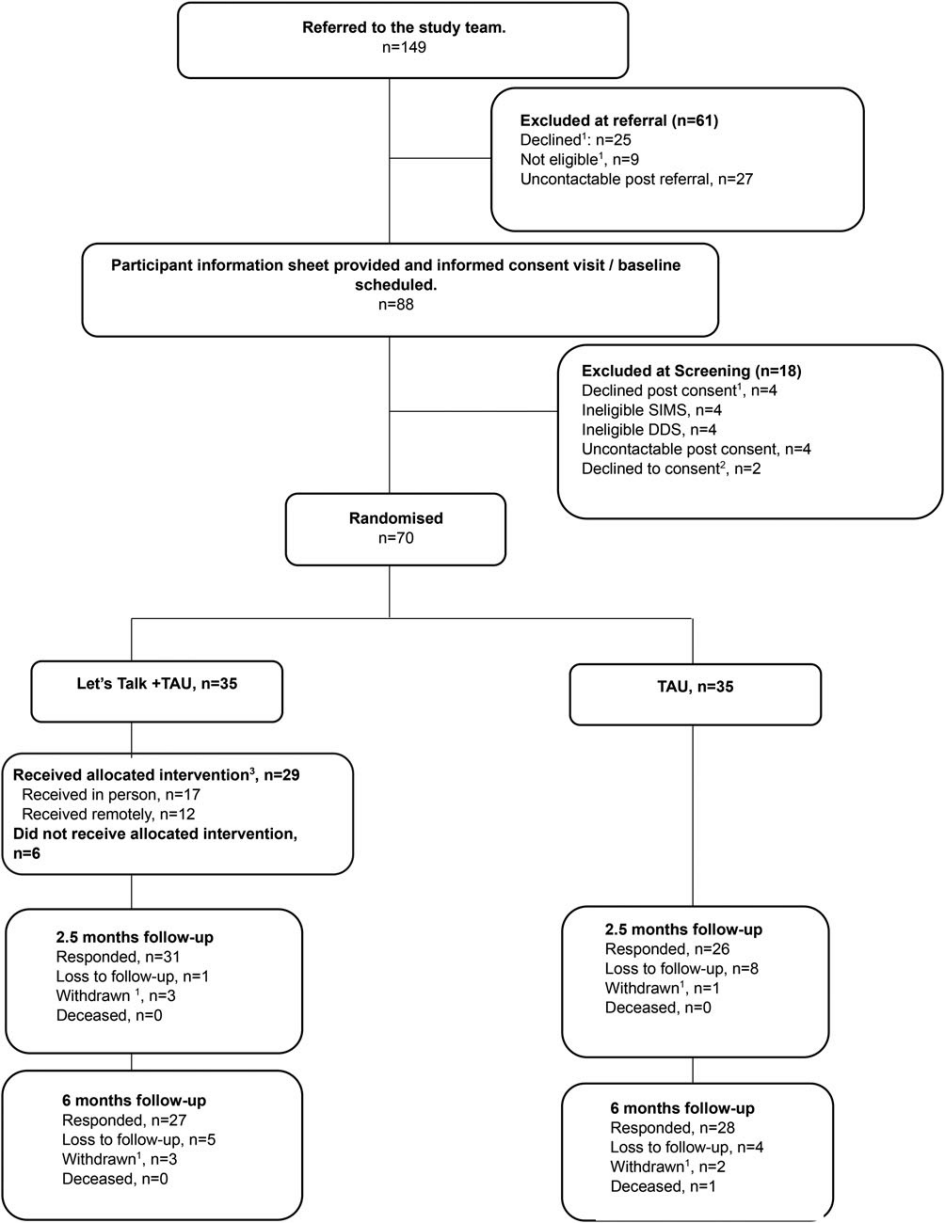
The CONSORT diagram. ^1^ reasons are shown in appendix p.36; ^2^ no reason provided; ^3^ defined as ⩾2 sessions attended. .

Between 1 September 2021, and 31 January 2023, 149 patients were referred to the study. The first participant was recruited on 20 October 2021, and the last on 31 January 2023, and the final follow-up data was collected on 19 July 2023. Of the 88 patients screened, 70 participants were randomized, 35 to intervention and 35 to TAU (Fig. 1). Baseline participant characteristics are shown in Table 1 and were balanced between groups. The mean age at baseline was 36.5 (s.d. 12.4) in intervention and 38.7 (17.4) in the TAU arm. In the intervention arm, 21 participants (60%) were male, with 17 males (49%) in TAU.

**Table 1.** Baseline characteristics

	Let's Talk + TAU *N* = 35	TAU *N* = 35
Age (years)	36.5 (12.4); 35	38.7 (17.4); 35
Gender – *n* (%)		
Male	21 (60.0)	17 (48.6)
Female	13 (37.1)	18 (51.4)
Nonbinary	1 (2.9)	–
Highest level of education – *n* (%)		
Higher	11 (31.4)	9 (25.7)
Further	10 (28.6)	15 (42.9)
Secondary	13 (37.1)	10 (28.6)
Primary	1 (2.9)	1 (2.9)
Employment status – *n* (%)		
Unemployed	23 (65.7)	22 (62.9)
Full-Time	3 (8.6)	4 (11.4)
Part-time	2 (5.7)	4 (11.4)
University Student	4 (11.4)	2 (5.7)
College Student	1 (2.9)	3 (8.6)
Voluntary	1 (2.9)	–
Carer	1 (2.9)	–
Marital status – *n* (%)		
Single	31 (88.6)	25 (71.4)
Married	–	6 (17.1)
Divorced	2 (5.7)	1 (2.9)
Separated	1 (2.9)	2 (5.7)
Widowed	–	1 (2.9)
Cohabiting	1 (2.9)	–
Living arrangements – *n* (%)		
Alone	13 (37.1)	13 (37.1)
Parents	13 (37.1)	10 (28.6)
Other, Family	4 (11.4)	5 (14.3)
Friends	3 (8.6)	2 (5.7)
Partner	1 (2.9)	3 (8.6)
Other	1 (2.9)	2 (5.7)
Ethnicity – *n* (%)		
White	21 (60.0)	25 (71.4)
Black/ African/ Caribbean/ Black British	6 (17.1)	2 (5.7)
Asian	2 (5.7)	5 (14.3)
Mixed/ Multiple ethnic groups	5 (14.3)	1 (2.9)
Other	1 (2.9)	2 (5.7)
Religion/belief – *n* (%)		
Atheism	12 (34.3)	13 (37.1)
Christianity	14 (40.0)	11 (31.4)
Islam	3 (8.6)	5 (14.3)
Judaism	–	3 (8.6)
Sikhism	–	1 (2.9)
Other	6 (17.1)	2 (5.7)
Disclosure stress scale	5.8 (1.1); 35	5.8 (1.2); 35
The process of recovery questionnaire	29.9 (14.6); 27	31.5 (12.0); 29
The Calgary depression scale	9.9 (4.9); 29	11.1 (6.0); 29
Manchester Short Assessment	49.2 (16.2); 20	52.2 (11.2); 23
EQ-5D-5L	0.455 (0.380); 18	0.511 (0.339); 21
Social Interaction Anxiety Scale	37.8 (22.9); 21	40.1 (15.2); 25
Rogers Empowerment Scale		
Total	95.3 (13.9); 20	92.9 (11.1); 18
Optimism and Control Over Future	10.2 (3.0); 21	9.6 (2.4); 20
Self-esteem and self-efficacy	23.2 (6.5); 21	22.7 (6.9); 19
Power	44.2 (3.1); 20	43.8 (3.0); 20
Community activism and autonomy	9.0 (3.3); 21	8.8 (2.2); 19
Righteous anger	19.4 (2.3); 21	20.1 (2.5); 20
Semi-structed Interview Measure for Stigma		
Total	23.2 (5.7); 35	22.2 (4.1); 35
Internalized stigma total	18.5 (4.5); 35	18.1 (3.8); 35
Perceived stigma total	2.7 (0.8); 35	2.6 (0.7); 35
Experienced stigma total	2.0 (1.2); 35	1.6 (1.1); 35
Stigma Stress Scale		
Total	0.6 (7.7); 28	2.5 (7.6); 30
Perceived harm	20.5 (5.2); 29	21.4 (5.1); 30
Perceived resources	20.3 (4.7); 28	18.9 (5.7); 30
Self-esteem rating scale		
Total	76.8 (26.2); 17	76.1 (24.4); 17
Positive	38.6 (16.4); 17	38.6 (11.8); 18
Negative	43.4 (13.5); 18	42.1 (13.7); 17
Internalised Shame Scale		
Total shame	88.3 (20.5); 18	83.0 (25.7); 16
Total self esteem	16.9 (7.2); 18	16.2 (5.5); 17

Data are *n* (%); mean (s.d.).

### Feasibility and safety outcomes

Our recruitment rate was 93% of the target sample size of 75 participants. Referral to randomization rate was about 2:1, with 25/149 (17%) referred individuals declining to take part, and two (1%) declining to consent (appendix p. 36). Referrals were slightly higher from Early Intervention in Psychosis services (91; 61%).

At end of treatment (2.5-month follow-up), 57 (81%) participants were retained for the semi-structured interview measure of stigma and 52 (74%) for stigma stress (Fig. 1). There were seven masking breaks (i.e. randomly assigned group revealed); six in the intervention arm and one in TAU.

Of those allocated to intervention, 29/35 (83%) received at least two sessions, which was defined as the minimal amount to meet adherence. Of those who allocated to the intervention arm 23/35 (66%) attended at least half of the total number of sessions available with a median of seven [IQR 2–8] sessions attended. Reasons for attending less than half the sessions available can be found in appendix (p.37). Median time to first session from randomization was 16 days [IQR 13–22], and 16/33 participants (48%) received a booster session. Full details of treatment received are in Table 2. Details of the peer fidelity index across all sessions and hospital use are shown in (appendix p.38)

**Table 2.** Treatment received and session data for those allocated to Let's Talk plus TAU

	All *N* = 35	Mode of delivery	
		In-person	Remote
Received their allocated intervention (⩾2 sessions)			
Yes	29 (82.9)	17 (85.0)	12 (80.0)
No	6 (17.1)	3 (15.0)	3 (20.0)
Number of sessions attended			
Zero	2 (5.7)	–	2 (13.3)
One	4 (11.4)	3 (15.0)	1 (6.7)
Two	6 (17.1)	4 (20.0)	2 (13.3)
Five	1 (2.9)	–	1 (6.7)
Six	5 (14.3)	3 (15.0)	2 (13.3)
Seven	7 (20.0)	4 (20.0)	3 (20.0)
Eight	9 (25.7)	5 (25.0)	4 (26.7)
Ten	1 (2.9)	1 (5.0)	–
mean (s.d.); *n*	5.5 (2.8); 33	5.4 (3.0); 20	5.8 (2.5); 13
median [IQR]	7 [2, 8]	6.5 [2, 8]	7 [5, 8]
Time to first session from randomization (days)			
mean (s.d.); *n*	18.8 (9.6); 33	19.8 (10.0); 20	17.4 (9.1); 13
median [IQR]	16.0 [13.0, 22.0]	16.5 [12.5, 27.0]	15.0 [13.0, 16.0]
Strategies completed			
Getting to know each other/ rapport building	32 (97.0)	19 (95.0)	13 (100.0)
Language, beliefs and behaviors about mental health	27 (81.8)	15 (75.0)	12 (92.3)
Identifying and challenging internalized stigma	27 (81.8)	16 (80.0)	11 (84.6)
Benefits and costs	23 (69.7)	14 (70.0)	9 (69.2)
Choices and settings for disclosure	23 (69.7)	13 (65.0)	10 (76.9)
Anticipating and managing how others may responding	23 (69.7)	12 (60.0)	11 (84.6)
Telling your personal story	21 (63.6)	12 (60.0)	9 (69.2)
Future directions	18 (54.5)	11 (55.0)	7 (53.8)

Values are numbers (percent) unless otherwise stated.

The as-treated analysis of safety, defined as attendance of at least one session of the intervention, are reported in Table 3. Safety by intention-to-treat is presented in appendix p.46. As-treated analysis showed 7/33 participants in intervention (21%, 14 events) and 6/37 in the TAU arm (16%, 9 events) had either a serious adverse event (SAE) or an adverse event (AE). For SAE's, 6/33 participant (18%, 9 events) in intervention and 3 participants (8%, 6 events) in the TAU arm. There was one death in the TAU arm due to a physical condition. Of the 15 SAEs, one event categorized as potentially life-threating self-harm, was deemed partially related, the participant and care team reported the event occurred following an increase in distressing psychosis symptoms, but that some of the research assessment questions were upsetting. This occurred on one occasion (at baseline) and the participant agreed to complete all subsequent assessments with no further adverse events in relation to the assessment. Across the 182 completed assessments the incident of SAEs arising from the assessment questions was 0.5%. For the AEs, all events were expected and unrelated.

**Table 3.** Serious and adverse events by treatment received

	Let's Talk + TAU *N* = 33	TAU *N* = 37
Number of participants with an SAE/AE	7 (21.2)	6 (16.2)
Number of SAE/AE	14	12
Serious adverse events		
Number of participants with an SAE	6 (18.2)	3 (8.1)
Number of SAE's	9	6
Details		
Admission to a psychiatric hospital (voluntary)	2	2
Potentially life-threatening self-harm^a^	1	0
Suicide attempt^b^	1	3
Admission to a psychiatric hospital (involuntary)^c^	2	–
Serious violent incident (participant as victim of incident)	2	–
Serious violent incident	1	–
Death^d^	–	1
Adverse events		
Number of participants with an AE	3 (9.1)	5 (13.5)
Number of AE	5	6
Details		
Increase in suicidal ideation	1	3
Increase in suicidal ideation and behaviors	2	–
Increase in suicidal ideation requiring intervention	1	–
Self-harm	–	1
Other	1	2

Values are either n (percent) or n.

Treatment received is defined by at least one session of the allocated intervention.

a This occurred post randomization but before the first intervention session.

b The event occurred before randomization.

c One event occurred post consent but before completion of a baseline assessment, participant later completed the assessment and was allocated to intervention.

d Physical health condition.

### Secondary outcomes

**Figure 2.**
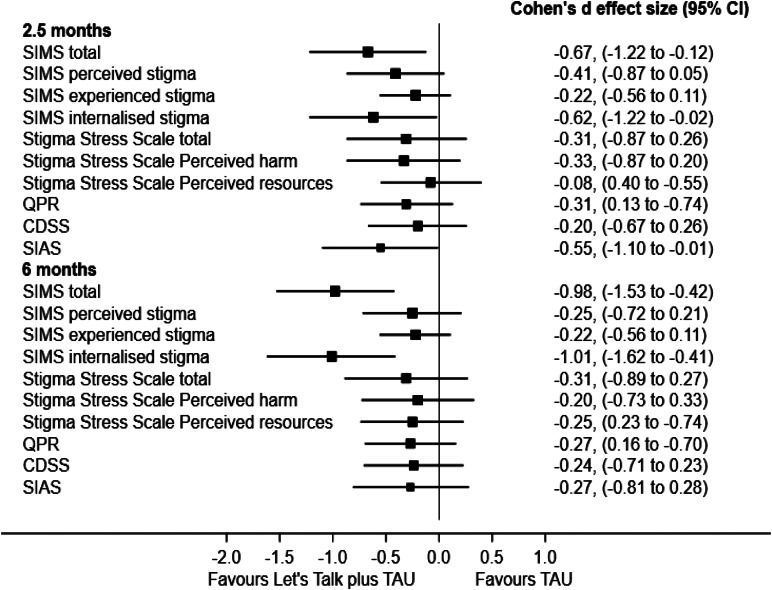
Forest plot of effect sizes Cohens d.

All secondary outcomes are reported fully in the appendix (pp. 38–39). The intervention was beneficial for one of the candidate primary outcomes at end of treatment; for total SIMS the mean difference (MD) was −3.31 (95% CI −6.03 to −0.59) favoring the intervention. For stigma stress total the MD was −2.33, (95% CI −6.65 to 1.99), favoring intervention. Results were similar at 6-months. Figure 2 shows the effect sizes for the two candidate primary outcomes of total SIMS and stigma stress, and other key secondary outcomes of personal recovery, depression, and social anxiety.

## Discussion

To our knowledge, this is the first feasibility RCT of individually delivered HOP for people with psychosis. Recruitment was feasible and attrition was low (<20% at end of treatment; 21% at 6-month follow-up). Completion rates suggest that the SIMS is a feasible outcome measure, and we propose it confers the advantage of assessing all three dimensions of personal stigma whilst engaging a participant in a meaningful conversation regarding their stigma experiences. Finally, uptake of the intervention was high. Taken together, the findings indicate feasibility for a future, larger trial and we make full recommendations for the design a future trial in Table 4.

**Table 4.** Lessons learnt, challenges faced and recommendations for a definitive trial: trial feasibility data

Activity	Lessons learnt/ challenge faced	Recommendation
Trial set-up		
Employment of peer support workers	Delays in staff can occur where sites do not have established research peer support worker roles including job descriptions approved by the host organization that are suitable for the intervention delivery i.e., that specify parameters regarding level of previous experience, peer support training/ qualifications. Delays can occur where job evaluation to assign pay scale is required. Delays in staff can impact recruitment to target in the early phase of the project.	Site selection should include review of peer role pathways to ensure there are appropriate and established job descriptions at a suitable pay banding. Job descriptions to be designed to reflect the role is not entry level peer support due to the nature of delivering a manualized peer intervention.
Trial integration with clinical services	Relationships are required with clinical services to ensure recruitment targets can be achieved from the start of the recruitment window. Building relationships can take time.	Each site requires involvement from an identified clinical lead in the relevant community services. Recruitment plans should include liaison and a recruitment launch for clinical services ahead of the recruitment window starting with input from key people with lived experience (PWLE) and clinicians.
Determining recruitment targets	Our data indicates that a recruitment of 4 per month is feasible.	Recruitment targets should be set at 4-per month.
Determining recruitment targets	Initial delays in recruitment can be experienced whilst the trial becomes established at site level.	A larger trial should consider a recruitment trajectory that accounts for initial delays in the study becoming established with clinical services and recruitment pathways.
Trial integration with voluntary and third sector services.	Recruitment focused solely on NHS services, which may have missed a recruitment source.	Recruitment strategy to include liaison with voluntary and third sector services.
Recruitment strategy	In our study, liaison with clinical services included raising awareness of stigma and discrimination, and how to recognize this in people with experience of psychosis. Overall, ineligibility rates at referral and screening were low suggesting efforts to raise awareness regarding how personal stigma manifests were helpful to recruitment/ referral pathways.	Recruitment strategy should include providing educational resources to clinical services and or third sector organizations that raise awareness of stigma, discrimination, and the impact it can have for people with psychosis. Such educational approaches should be co-produced by people with lived experience (PWLE) of psychosis.
Recruitment strategy	A relatively high number of service users referred were not contactable post referral.	Establishing strong relationships with clinical services and care coordinators to support contact post referral are likely to reduce the number unable to contact post referral. Patient and Public Involvement groups should be consulted to identify suitable approaches to initial contact.
Trial design		
Stigma related primary outcome	Our trial data demonstrates that it is feasible to collect both proposed primary outcomes for a definitive trial. Response rates for the Semi-structured Interview Measure for Stigma in Psychosis (SIMS) were in the green progression zone suggesting feasibility for a definitive study, without further modification to data collection. Response rates for the stigma stress questionnaire were in the amber zone, suggesting that some adaptations to data collection is required if this measure was selected as the primary outcome. It is likely stigma stress return rates were slightly lower than SIMS due to the priority given to SIMS, which was favored by our participatory/ patient and public involvement group.	As a stigma primary outcome for a lager efficacy trial, the SIMS is recommended. This measure is interview based, allowing for engaging and meaningful conversations regarding personal stigma, was prioritized by our participatory/ patient group, and had response rates in the green zone.
User-defined recovery as primary outcome.	Our trial data shows a small effect size for user-defined recovery at end of treatment and an improvement on user-defined recovery of 4-points, which is considered to be the Minimal Important Difference of the user-defined recovery scale.	A larger trial of efficacy may choose to adopt a primary outcome that tests the efficacy of the intervention for a broader wellbeing outcome. User-defined recovery as assessed by the Questionnaire about the process of recovery scale (QPR) is recommended as a primary outcome for a larger trial of efficacy.
Intervention delivery: treatment envelope	On average, it took at least 2.5 weeks for the first session to take place and some of the latter workbook sessions were less frequently completed. The primary reason for receipt of less than half the available sessions was non-attendance or cancellation of sessions, limiting total number available in the treatment window.	A longer intervention window would allow sufficient time for first appointments, which can be delayed when there are challenges with making initial contact (participants not responding to phone calls or text messages), and part-time working hours of peers (which is commonplace). A longer intervention window would also ensure sufficient time to complete the full complement of workbook strategies. We recommend a treatment envelope of four months.
Intervention delivery: workbook strategies	Rates of completion were slightly lower for workbook strategies of costs and benefits of disclosure, choices, and settings for disclosure, developing a personal narrative for disclosure, testing who is a good person to disclose to, and future directions. Our session record data does not indicate why these strategies were lower, although we hypothesize this may in part be related to the treatment envelope of 10-weeks. It is also possible these exercises may have been declined by participants. However, it is not possible to determine the reason without session record data.	A future trial should include in the session records reason for non-completion of planned strategies. This will allow ongoing review during the trial and where required offer support for any training needs through supervision.
Intervention delivery: booster session	50% of participants received a booster session.	Boosters should be retained and offer of booster monitored within supervision. Reasons why boosters are not completed should be captured on session records.
Intervention delivery: assertive outreach approach	Peer support workers took an assertive outreach approach to delivery and uptake of the intervention was good with 87.9% receiving at least two sessions and the average number of sessions as 5.5.	Delivery should retain an assertive outreach approach to ensure maximum opportunity for engagement with the intervention.
Intervention delivery: peer support worker supervision	Supervision from both a peer specialist and clinician were complimentary and allowed both review of peer principles and clinical requirements of working as a PSW in statutory settings. Group format of supervision ensured peer-to-peer connection between the PSW, which may be even more indicated in a definitive multi-site study.	PSW supervision should include a peer specialist, clinician and be in a group format.
Methods to maintain the blind	Blind breaks were more common in the intervention arm and the presence of the workbook a threat to the blind.	PSW should prompt/ remind participants about the blind at end of intervention and prompt that the workbook should be kept out of view when the follow-up is completed with the research assistant.
Suitability of randomization procedures	As randomization was performed by a concealed web-based platform developed by the Clinical Trials Unit, the risk of selection bias was low. The centralized web-based platform hosted by the CTU was suitable for use in a definitive trial	A web-based platform for randomization, that is centralized with a Clinical Trials Unit platform should be used to randomize participants and reduce the risk of selection bias.
Comparator	No active control	A future trial should consider enhanced treatment as usual/ control arm. Psychoeducation in the form of information provision (this could be written or online) may provide a suitable active control.
Burden of assessments	Low response rate to many of the secondary outcome measures suggesting the assessment battery was burdensome.	A reduce assessment battery is required for a definitive study. The assessment battery should contain only essential measures to answer the primary and secondary research question and hypotheses, this may include measures for mediation or moderation where required. Furthermore, the assessment pack should be informed by Patient and Public Involvement, specifically regarding burden and potential for impact on distress.
Cost-effectiveness	Service use data and EQ-5D-5L had a low response rate.	Strategies for collecting service user data required for a cost-effectiveness analysis should be considered. Strategies that link with extraction of data from Electronic Patient Records would reduce burden and reduce risk of memory bias from self-report.
Retention to follow-up	Attrition was somewhat higher in the TAU group.	Strategies to demonstrate that participants in both arms of the trial are valued may balance attrition across arms. This could include maintaining contact with participants in both arms at points between baseline and follow-up's by sending a thank you card and a study newsletter. Patient and Public Involvement groups should be consulted on ways to maintain engagement in the study.

Our trial took a rigorous approach to adverse event monitoring and as-treated SAE data shows nine SAEs in the intervention group *v.* six in the control. However, two SAEs in the intervention group took place before randomization and one event was prior to first intervention session and no intervention had been received at the time of event. As such, our adverse event data indicates that the adaptation of HOP from group to individual delivery was safe.

The most delivered intervention strategies were establishing the peer relationship, developing a shared understanding of stigma and mental health identity, and challenging IS beliefs. Around two thirds of participants allocated to the intervention received the strategies of: benefits and costs of disclosure, choices, and settings for disclosure, anticipating and managing how others may respond to disclosure, and personalized approaches to sharing psychosis experiences. Broadly, self-rated fidelity scores indicate the intervention was consistent with principles of peer support (Gillard et al., 2021). Principles most frequently endorsed were mutuality, reciprocity, and valuing experiential experience. Our data suggests minimal difference between in-person or remote delivery for completion of workbook strategies, or delivery consistent with peer principles.

Our preliminary data on the clinical effects of Let's Talk for stigma show moderate effect sizes at end of treatment for IS and total stigma, and large effect sizes for IS and total stigma at 6-month follow-up. Group HOP has been shown to have a small effect size for IS (Rüsch & Kösters, 2021). Our findings regarding stigma stress broadly align with the literature as we observed a small effect size for stigma stress at end of treatment and follow-up. HOP trials have demonstrated a moderate effect size on stigma stress at end of treatment and a small effect size on stigma stress at 3–4-week follow-up (Rüsch & Kösters, 2021).

Regarding the potential clinical effects on user-defined recovery (Law et al., 2014) our data show a small effect size at end of treatment on the QPR which aligns with the literature regarding the effects of peer support on personal recovery (White et al., 2020). The Minimal Important Difference (MID) for the QPR is a four-point increase on the scale (Dehmahdi et al., 2021). We observed a mean difference of four points at end of treatment, indicative of clinically meaningful benefit for user-defined recovery that is worth evaluating in a larger trial.

Whilst our trial confirms it is feasible to use the SIMS as a primary outcome in a future trial, the observed effects of the intervention on user-defined recovery are noteworthy and may suggest that recovery is an appropriate primary outcome for a future trial. The absence of assessment of this important outcome by existing IS intervention studies has been recognized (Thornicroft et al., 2022) and both PLWE of psychosis and UK national guidelines consistently prioritize user-defined recovery as a core outcome for people with psychosis (National Institute for Health and Care Excellence, 2014). A definitive trial should embed mediation and moderation tests to evaluate the mechanisms of change and if recovery was elected as a primary outcome a candidate mechanism of change would be IS, which has been shown to be negatively related to recovery (Eliasson et al., 2021).

The primary limitation is that the study was not powered to detect clinical effects and results should therefore be interpreted cautiously. The primary challenge to delivery was covering the full workbook content in the allocated 10-week intervention window. In some cases, we experienced a delay in time to first session and the key driver for attending less than half the available sessions was non-attendance/cancellation of sessions by the participant. We recommend a future trial extend the number of sessions available and provide a longer intervention window. Our study does not demonstrate which elements of the intervention are most effective, or whether the intervention confers more benefit for specific groups, and a future trial should consider a process evaluation to address these questions. TAU for participants included care from a mental health team and so it is not possible to exclude the potential for any observed benefits to be attributable to support offered within these services, although it is important to note that both arms of the study received TAU. Whilst 16% of referred participants declined an informed consent visit and 18% were uncontactable after referral, we consider this a result of the broad *v.* targeted approach to recruitment taken for this study. Based on service user consultation we did not include a measure of psychotic experiences; this may limit the ability to determine the relationship between psychosis symptoms and stigma or describe symptom severity in the study population. The SIMS has been used in one other stigma intervention trial (Morrison et al., 2016) and this may limit comparison to other stigma intervention studies; other self-report stigma measures are available. Finally, we did not formally assess the practicality of the intervention (Bowen et al., 2009).

Given the demonstration of feasibility and encouraging observed reduction in IS and increase in user-defined recovery we conclude that a definitive test of efficacy or effectiveness of the Let's Talk intervention is required.

## Supporting information

Pyle et al. supplementary material Pyle et al. supplementary material

## Figures and Tables

**Table:** 

Annabelle	Durrad
Safiya	Adams
Cláudia	Gonçalves
Corey	Lee
Alana	Potter
Rose	Stevens-Neck

## Supplementary material

The supplementary material for this article can be found at https://doi.org/10.1017/S0033291724002605

## Data sharing

Deidentified participant data will be available with publication in anonymized form from the corresponding author (melissa.pyle@gmmh.nhs.uk) on reasonable request, subject to review and contract with Greater Manchester Mental Health NHS Foundation Trust.
