# Supplementary material for: A feasibility randomized controlled trial of an individually delivered, peer support intervention to reduce the impact of psychosis stigma and discrimination for people with psychosis: the let's talk study
Source: Psychol Med. 2024 Dec 27;54(16):4600–11. doi: 10.1017/S0033291724002605 (PMC11769902; doi:10.1017/S0033291724002605)
Supplement: Pyle et al. supplementary material [file S0033291724002605sup001.docx]

**A feasibility randomised controlled trial of a peer-delivered intervention to reduce the impact of psychosis stigma and discrimination for people with psychosis: The Let’s Talk Study**

**Appendix**

**Contents**

[**Study Protocol** 2](#_Toc175683275)

[**Intervention details** 21](#_Toc175683276)

[**Fidelity Scale** 24](#_Toc175683277)

[**Results** 36](#_Toc175683278)

**Tables**

[**Table 1: Participant exclusions** 36](#_Toc175683279)

[**Table 2: Reasons for change of status** 36](#_Toc175683280)

[**Table 3: Referral pathway** 37](#_Toc175683281)

[**Table 4 : Reasons for receiving less than half the sessions available.** 37](#_Toc175683282)

[**Table 5 Secondary outcomes** 38](#_Toc175683283)

[**Table 6: Peer Fidelity Index across all sessions** 41](#_Toc175683284)

[**Table 7: Hospital use at Baseline** 42](#_Toc175683285)

[**Table 8: Hospital use at 2.5 months follow-up** 43](#_Toc175683286)

[**Table 9: Hospital use at 6 months follow-up** 44](#_Toc175683287)

[**Table 10: Serious and adverse events by allocation** 46](#_Toc175683288)

# **Study Protocol**

**v 7.0 17/NOV/2022**

**Full project title:** A peer-delivered intervention to reduce the impact of psychosis stigma and discrimination: a feasibility Randomised Controlled Trial

**Short project title: ‘**Let’s Talk’: a feasibility Randomised Controlled Trial

**IRAS Number:** 300859

**Sponsor Reference number** x538s

**REC Number:** 21/SC/0232

**NIHR Number:** NIHR 200460

**Sponsor details:** Greater Manchester Mental Health NHS Foundation Trust

**Chief Investigator details:** Dr Melissa Pyle

**CI Address:** The Psychosis Research Unit, Greater Manchester Mental Health NHS Foundation Trust, Harrop House, Prestwich, M25 3BL

**CI email address:** [melissa.pyle@gmmh.nhs.uk](mailto:melissa.pyle@gmmh.nhs.uk)

**Background and Rationale**

Psychosis is a mental health problem including experiences such as hearing voices and paranoid beliefs, cognitive disorganisation and negative symptoms (e.g., social withdrawal). Stigma is defined as “an attribute that is deeply discrediting” which turns a person from “a whole and usual person to a tainted, discounted one” (Goffman, 1963). A commonly reported and problematic consequence of stigma is internalisation of stigmatising beliefs towards oneself, referred to as internalised stigma (IS). Around 40% of people with a schizophrenia diagnosis report at least moderate level of IS (Brohan et al., 2010). Research indicates that IS has a long-term effect on service user defined recovery as it can worsen symptoms of psychosis (Vass et al., 2015), cause emotional distress (Rüsch et al., 2009a; Rüsch et al., 2009b), depression (Upthegrove et al., 2017) and anxiety (Alonso et al., 2011), decreased self-esteem (Vass et al., 2015), increase hopelessness (Watson et al., 2007) and result in poorer functioning (Hill et al., 2013). The personal impact of psychosis because of stigma including loss of social role, shame and internalisation of stereotypes has been associated with depression in psychosis (Birchwood et al., 1993; Upthegrove et al., 2017).

As mental health problems are not visible and because of stigma and discrimination, those who experience psychosis and other mental health difficulties face a difficult decision about whether to disclose their psychosis diagnosis, which can for some people result in secrecy, shame and social withdrawal (Vauth et al., 2007; Corrigan et al., 2013). Psychosis is one of the most stigmatised mental health conditions (Angermeyer & Matschinger, 2003) so decisions about disclosure are particularly difficult.

Tackling mental health stigma is a current UK government priority. In the recent “Five Year Forward View for Mental Health” (Taskforce, 2016), one of six priorities for research is reducing stigma and empowering people with mental health problems. The NICE Guideline CG178 recognises the problem of stigma for people with psychosis and emphasizes the importance of recovery-orientated services and recovery as an outcome for service users (NICE, 2014).

One approach to addressing IS that shows promise is peer support (PS). A recent meta-analysis indicates that group PS interventions have a small but significant effect for improvements for self-efficacy and empowerment (Burke et al., 2018). In addition, it has been argued that delivery of interventions to reduce IS by mental health professionals could unintentionally pathologise this as a problem located in the individual, rather than as an understandable reaction to a social injustice (Corrigan, 2016). Therefore, peers may be best placed to address IS and its harmful consequences in people with psychosis (Corrigan, 2016). The Schizophrenia Commission identified that service users with psychosis and schizophrenia value PS and recognised that interventions such as PS can address stigma and discrimination. The “Five Year Forward View for Mental Health” includes PS in its vision for the future of mental health services, emphasising support for paid PSW roles (Taskforce, 2016). One peer-led approach that has shown promise is the Honest Open and Proud (HOP) programme, which provides an opportunity for patients to discuss mental health stigma and explore their disclosure decisions with peers. HOP offers an opportunity to consider and make decisions about disclosure and provides access to a peer support worker (PSW). HOP was developed to address the key mediators of IS and its harmful impact, with a specific focus on disclosure [3]. There are three pilot RCTs supporting the efficacy of HOP (Corrigan et al., 2013; Rüsch et al., 2014; Mulfinger et al., 2018) in the US and Germany. They found reductions in stigma stress, disclosure stress and desire for secrecy for the HOP groups compared to control groups at end of treatment and follow-up. HOP has also been shown to reduce depression (Corrigan et al., 2013; Mulfinger et al., 2018). A recent systematic review and meta-analysis of HOP interventions has shown small but significant effects for stigma stress and small but non-significant effects for internalised stigma (Rüsch & Kösters, 2021). The review also identified recruitment of participants as a challenge faced by two HOP trials with the authors hypothesising the group delivery of the intervention as a potential barrier to participation in the trial. There are many barriers to implementing groups for people with psychosis, including fears regarding stigma and discrimination, poverty, social exclusion, and social anxiety and resulting avoidance and practical issues such as participants being geographically dispersed. These factors that present challenges to regular group attendance have resulted in previous attempts to deliver group-based interventions to people with psychosis being unsuccessful, even in the context of a preference trial (Morrison et al., 2006). In addition, trials conducted to date have had small sample sizes and no follow-up beyond month.

HOP has recently been modified through Public and Patient Involvement (IRAS application: 200460) to adapt for delivery by NHS peer support workers in a one-to-one setting (rather than a group setting) for people with experience of psychosis living in the UK. As part of the modification work, the name HOP has been changed to Let’s Talk and the peer-delivered intervention shall be referred to as such throughout this protocol. Modifications were informed through review of the HOP workbook and manual with service users who have experience of psychosis and peer support workers. In addition, wider PPI review was obtained through consultation with the Psychosis Research Unit Service User Reference Group and co-applicants with lived experience of psychosis and peer support.

The research carried out under IRAS application 200460 and the research outlined within this protocol is funded by the National Institute for Health Research (NIHR), Research for Patient Benefit (RfPB).

**Study aims and objectives**

The principal objective of the proposed research is to evaluate the feasibility and acceptability of conducting a randomised controlled trial (RCT) of the ‘Let’s Talk’ peer-delivered intervention with people who experience psychosis.

Specific objectives for trial are to assess:

1. The proportion of eligible people clinicians are willing to refer, the proportion of eligible people willing to participate, the proportion of participants who engage with the intervention
2. The proportion of participants who select in-person vs. remote delivery of the Let’s Talk peer-delivered intervention.
3. The drop-out rate.
4. The characteristics of trial participants to refine selection criteria.
5. The appropriateness and integrity of Let’s Talk workbook and manual.
6. The feasibility and acceptability of the intervention to participants.
7. The randomisation procedures.
8. The relevance (to participants) and validity of the measures to assess effectiveness, service use, health status, safety and acceptability in a subsequent definitive trial.

**Study Design**

We will conduct a feasibility randomised controlled trial (RCT) across two UK sites, Greater Manchester, and Northeast London. Participants who meet the inclusion criteria will be randomly allocated to either Let’s Talk plus treatment as usual (TAU), or TAU alone.

We will randomise participants at the individual level using an independent remote web-based randomisation system produced by The Centre for Healthcare Randomised Trials (CHaRT) Clinical Trials Unit (CTU), using randomised-permuted blocks of random size. Randomisation will be in 1:1 ratio stratified by site and delivery mode (in person or remote).

The research assessments will be conducted by raters (blind to allocation) at baseline, end of treatment (2 months) and 6 months post-randomisation.

Green progression criteria to a definitive trial would be likely to include:

1. Recruitment rate within ≥80% (green); 79-60% (amber); <60% (red) of planned target.

2. Retention of participants within the study with ≥ 80% (green); 79-60% (amber); <60% (red) of data for proposed primary outcome of a definitive trial.

3. ≥ 80% (green); 79-60% (amber); <60% (red) of those allocated to Let’s Talk receiving at least two sessions.

We will conduct a nested qualitative study to explore the subjective experience of receiving or delivering the peer-delivered intervention ‘Let’s Talk’, elicit service user views of adverse effects and benefits, and identify themes relating to these issues. This will involve individual interviews with a purposive sample of 15/20 service users who have been randomised to receive Let’s Talk. We will purposively sample individuals with differing levels of engagement with Let’s Talk. Interviews will explore participants’ expectations and experiences of the intervention and the research trial including recruitment, consenting and randomisation. Qualitative interviews will also be conducted with PSWs delivering Let’s Talk to explore their experiences of the role, from training, supervision to delivery. Qualitative data from participants and PSW will provide an opportunity for further iterative work to refine and develop Let’s Talk and trial processes to maximise their acceptability in a definitive trial.

**Methods**

**Participants**

Inclusion will be as follows:

1. Age 16+
2. Meet ICD-10 F20-F29 Schizophrenia spectrum diagnosis, or be receiving services from Early Intervention Services (EIS)
3. Under the care of a mental health service with a care coordinator
4. Competent to provide written, informed consent (for ethical considerations).
5. At least a moderate level of self-reported disclosure-related distress screening scale (Rüsch et al., 2014), as determined by scoring >3 on the screening item.
6. At least moderate levels of internalised stigma as determined by a score of ≥3 on at least one of the Internalised Stigma domains the Semi-structured Interview Measure of Stigma (Wood et al., 2016)

Exclusion criteria will be as follows:

1. A primary diagnosis of alcohol or substance dependency (ICD-10 F10 – F19 diagnosis), where this is clearly the cause of their psychotic symptoms. This does not exclude people who use substances or alcohol, only those with a primary diagnosis. This will be confirmed by the participants care coordinator or psychiatrist.
2. A diagnosis of moderate to severe learning disability, as confirmed by the participants care coordinator or psychiatrist.
3. An ICD-10 diagnosis of organic psychosis, as confirmed by the participants care coordinator or psychiatrist.
4. Non-English speaking, where this prevents engagement in informed written consent and interviews.
5. Immediate risk to self or others as confirmed by the participants care coordinator or psychiatrist.

**Intervention**

Let’s Talk is an adaptation of the Honest, Open and Proud (HOP) program that comprises of a workbook and manual for the delivery of the workbook and is delivered in a group format by one or more facilitators trained in the manual and typically at least one facilitator has with lived experience of a mental health problem (Corrigan et al., 2013; Rusch et al., 2014; Mulfinger et al., 2018). Adaptations have been made to ensure localisation to the UK context and delivery in a one-to-one setting by a Peer Support Worker. HOP can be found here: <http://www.comingoutproudprogram.org/index.php/manual-and-resources>.

Let’s Talk will be delivered, in addition to TAU, on a one-to-one basis by a Peer Support Worker (PSW) with up to ten weeks of the Let’s Talk programme available. The ten-week time frame commences when the participant is randomised. It is excepted sessions will be weekly. The aims of Let’s Talk are to: help participants weigh pros and cons of disclosing which vary by setting (e.g., disclosure at one’s employment has different costs and benefits than disclosure to one’s friendship network); teach relatively safe ways to disclose should the person decide to do so; help people craft stories that reflect their disclosure goals; support participants with internalised stigma and developing affirming self-beliefs. All participants allocated to Let’s Talk will received a copy of the workbook based. Sessions with the PSW will be structured around the manual and workbook. Participants in GMMH NHS Foundation Trust will have the option to meet with the PSW either in person at a community venue agreed collaboratively, this could include the participants home, or by a remote method (telephone/ online) we will determine this at baseline assessment and randomisation will be stratified by delivery mode. Since the PSW will be employed and based at GMMH it will not be possible to offer an in-person delivery mode at North East London NHS Foundation Trust. Participants will have the option of a Let’s Talk booster session that will review disclosure decisions, use of peer support programs, current assessment of pros and cons of disclosure and whether the person’s story has changed. PSWs will have substantive posts with the NHS and will be based in the research team at the Psychosis Research Unit. PSWs will receive regular supervision from an experience PSW and a clinical psychologist. Clinical supervision will address specific NHS requirements such as managing risk and safeguarding, maintaining clinical records, and working in line with local NHS Trust policy. To check manual fidelity, we will use a checklist covering the Lets’s Talk workbook content that is adapted from previously used HOP fidelity scale [3,5,6]. To determine the feasibility of delivering the intervention in line with peer support principles we will also assess fidelity using the Principle Based Fidelity Index by Gillard et al. (2021).

With consent from the participant and the PSW, the Let’s Talk sessions will be recorded, and a random sample will be assessed for fidelity to the manual. Following each session, PSW will complete a session record that monitors content of sessions in terms relevant to the workbook and manual.

**Comparator**

The control condition is treatment as usual (TAU) which includes EIP and secondary care AMH services. All participants in the intervention and comparator arms will have access to one of these services and an identified care coordinator. Let’s Talk is not currently available as part of TAU at the recruiting sites. On exit from the trial, after the 6-month assessment is complete we will offer comparator participants the resource information from the Let’s Talk Workbook Appendix i.e., information regarding psychosis narratives provided in written and spoken word, information about local peer support groups and two leaflets ‘What is Stigma’ and ‘Myths and Facts about Psychosis’. We will not ask referrers to withhold any treatment. Research Assistants will identify any risks to self or others that require immediate action. Participants in both groups will receive a crisis card providing emergency contact details and all participants will have an allocated care coordinator. All routine or additional treatments in both conditions will be monitored.

**Outcomes**

As a feasibility RCT, a single primary outcome is not meaningful. The key outcomes to inform a definitive trial are referral rates; recruitment; attendance at the Let’s Talk sessions (number of sessions); fidelity to the Let’s Talk manual and principles of peer support and the most used Let’s Talk strategies; and follow-up/questionnaire response rates.

Outcomes measures will be collected to determine their suitability for use in a definitive trial and identify a suitable primary outcome for a definitive trial, rather than to draw conclusions about efficacy of treatments. Outcome measures will be collected at end of treatment (2.5 months) and 6 months post randomisation follow-up. In addition to demographics, we will collect the following outcomes:

1. The Semi-structured Interview Measure for Stigma in Psychosis (SIMS; Wood et al., 2016) which assesses experienced, perceived, and internalised stigma.
2. Disclosure related distress, a single item question rated on a 1-7 Likert Scale. (Rüsch et al., 2014)
3. Stigma stress will be assessed by the 8-item Stigma Stress Scale [Rüsch et al., 2009)
4. Recovery will be assessed using a service-user defined, 15-item self-report measure of recovery, The Process of Recovery Questionnaire (Law et al., 2014)
5. The Calgary Depression Rating Scale for Schizophrenia (CDSS50)), a valid and reliable interviewer-administered measure of depression. This scale is designed to minimise the potentially confounding symptom overlap between depressive features and both negative symptoms and extrapyramidal symptoms (Addington et al., 1990).
6. A measure of anxiety, Social Interaction Anxiety Scale (SIAS; Mattick and Clark, 1998).
7. Quality of life will be assessed by the Manchester Short Assessment of Quality-of-Life Scale (Priebe et al., 1999)
8. Empowerment will be measured using the Rogers Empowerment Scale (RES), a 28-item questionnaire with a 4-point scale from completely disagree to fully agree with a higher total score indicating a greater sense of empowerment (Rogers et al., 1997).
9. Help-seeking and service utilisation/engagement will be assessed by an economic patient questionnaire (Davies et al., 2008).
10. Health status will be evaluated using the EQ5D, which is a generic and validated health status questionnaire shown to have acceptable validity in people with schizophrenia in European countries (Bobes et al., 2005)

We will collect data on the below potential mediators of change to determine feasibility of collecting these measures in a definitive trial to answer important hypotheses about mediators of

1. Internalised Shame Scale, 30 item scale to measure internalised shame (Cook & David, 1997).
2. Self-Esteem Rating Scale - Short form, a 20-item measure of self-esteem (Lecomte et al., 2006).

Assessment of safety: The following will be recorded as serious adverse events (SAE): all deaths, suicide attempts, serious violent incidents, admissions to secure units, formal complaints about the intervention. Any SAE which is deemed to be related will be reported to the Research Ethics Committee (REC). The responsible clinical team, the trial management committee, the REC and the data monitoring committee (DMC) will be informed of any SAEs.

**Sample size**

We will recruit 75 participants and randomise in the 1:1 ratio. We need outcome data on 60 participants to estimate the standard deviation required for a sample size calculation for a definitive trial. We have used the advice from Whitehead et al. (2016) to plan ahead for a trial with 90% power and potential small-to-moderate effect size (standardised effect size ranging from 0.2 to 0.4). We have factored in 20% attrition on the primary outcome in this pilot study, the proportion of missing data is also a design parameter of interest for a future trial. A pilot study of this size will allow to test procedures and gather information on other trial design and process elements that are listed in the objectives section.

**Settings and recruitment**

The recruiting sites are Greater Manchester Mental Health (GMMH) NHS Foundation Trust and North East London NHS Foundation Trust (NELFT). Participants at the NELFT site will receive Let’s Talk remotely i.e., via telephone or an online platform such as zoom. We will recruit participants through several routes as outlined below.

We will identify potential participants via secondary care mental health services across the footprint of GMMH and NELFT these will include but are not limited to: Early Intervention in Psychosis (EIP) services, Adult Mental Health (AMH) services, inpatient settings where participants are due for discharge and the referring clinician considers it appropriate to approach the service user about the study, and psychology services. Research Delivery Team (RDT) staff from GMMH and NELFT based in secondary care services will work with clinicians to identify potential participants, obtain delegated first contact as per the GMMH/NELFT SOP, approach potential participants and seeking consent to refer details on to the research study team for them to follow-up. Clinicians/members of the RDT will seek permission from the potential participant to make a referral including sharing basic contact details and inclusion criteria information. To ensure an audit trail of this communication between the clinician and potential participant the service users responsible clinician will make a note in the potential participants NHS Health and Social Care Record to document when and by whom permission to refer was sought. The member of the research team taking the referral will check with the referring clinician that they have entered a note documenting permission to refer. Recruitment materials will include: a leaflet for NHS staff about the study and a leaflet for service users about the study for service waiting rooms/ staff rooms. These will be disseminated by the research team to all key NHS staff working in the target services and we will ask care coordinators to distribute these recruitment materials to potential participants. With the permission of the NHS Trust Communications Department we will also notify staff of the study via a desktop splash-screen and via the NHS Trusts Twitter accounts and provide a link via the Study Twitter account to [www.psychosisresearchunit.com](http://www.psychosisresearchunit.com) where the ethics approved study documents i.e. leaflets, and information sheet can be found. We will only use ethics approved content from the study leaflet and the PIS in this communication.

Clinicians in the above services will be made aware of the study via a number of routes: (1) the research team will meet with clinical teams to promote the study at team meetings or learning events, (2) via information about the study on our website [www.psychosisresearch.com](http://www.psychosisresearch.com) or via the study/ NHS Trusts Twitter account, (3) via information shared on NHS trust desktop display splash-screens, NHS Trust intranet pages. We will accept a referral from a researcher working on another study at the Trust who identifies a potential participant for this study. As with clinicians the researcher will seek permission from the potential participant to make a referral including sharing basic contact details and inclusion criteria information. To ensure an audit trail of this communication between the clinician and potential participant the clinician will make a note in the potential participants NHS Health and Social Care Record to document when and by whom permission to refer was sought.

We will accept self-referrals from people who have become aware of the study through viewing the study leaflet in the waiting rooms of the above NHS services and in relevant non-statutory/voluntary sector services located across the footprint of the above NHS trusts, or via information about the study on our website [www.psychosisresearch.com](http://www.psychosisresearch.com), or via the study/ NHS Trusts Twitter account. Self-referrals are the method adopted by current RCTs of HOP in the USA and in Switzerland. A person making a self-referral will be informed on first contact that we will make contact with their care coordinator to inform them of the self-referral (given care coordination is part of the inclusion) and seek verbal consent to do so.

In total we will recruit 75 participants over a 17-month recruitment window. We will recruit 65 of these participants from GMMH and 10 from NELFT. The lower recruitment rate in NELFT reflects the potential pool of eligible service users and that only the remote delivery option of the intervention will be available at this site. GMMH will be expected to recruit on average 4 participants per month over the 17-month recruitment window. NELFT will be expected on average to recruit 0.6 participants over the 17-month recruitment window.

All participants will be provided with a Participant Information Sheet (PIS) that complies with the requirements set out in ICH-GCP Topic E6 R1. To account for remote delivery approaches the information sheet will either be emailed to the potential participant or sent to them in the post. This will be provided to participants in advance of taking written informed consent and they will be given at least 24 hours to consider their participation. After signing the consent form participants will be free to withdraw participation in the interview at any point. However, we will retain the data we obtain. Recruitment will end on 31^st^January 2023.

**Study procedures by visit and safety reporting**

Full details of the interactions and visit for service user participants through the lifetime of the study are listed below in Table 1.

For all participants in NELFT, or for GMMH participants where national or local covid-19 restrictions prevent in-person visits, or for GMMH participants who do not wish to have in-person visits we shall offer remotely delivered appointments i.e., telephone or via an online platform.

Where an appointment is to be conducted remotely, the researcher or peer support worker will follow the below checklist:

- Check the individual’s access to sufficient mobile data or Wi-FI where relevant.
- Ensure the individual has knowledge of how to use any platforms/technology where relevant.
- Prior to consent, assessment or a Let’s Talk session confirm how the participant would like to receive relevant written information including post, email, or screen share. Check on the individual knowledge of how to use the technology if screen-sharing is preferable. Check on ability to read any emailed information i.e., does their technology support word documents/ PDF and is the screen size suitable for reading information.
- Check explicitly on the level of confidentiality of the individual’s environment. The assessment may need to be rescheduled if the participant is unable to speak openly.
- Check more generally that the environment is quiet and ideally be low risk regarding interruptions.
- Agree contingency plan if connection lost or technical issues arise. Including alternative contact if primary connection fails
- Agree plan for managing distress & risk: Collaboratively discussing and agreeing a plan for what the participant and research worker will do if the participant becomes distressed during the meeting. This must include that the research worker will contact the participant’s clinical team if the participant becomes distressed and terminates the call and the research worker cannot re-establish contact.

Where appointments are in-person the venue shall be agreed in collaboration with the participant and the researcher/ peer support worker will endeavour to meet the participant at a venue of their choice including the participants home or a community venue. The researcher or peer support worker will adhere to guidelines to reduce the transmission of covid-19 as specified by the government and NHS Trust that employs the researcher or peer support worker. The researcher and peer support worker will adhere to NHS Trust safe working policies and the Psychosis Research Unit safe working policy including completing a safety check prior to and on exit from each in-person visit.

| **Procedure** | **Details** |
| --- | --- |
| Introductory phone call and information about the study | On receipt of referral a member of the research team will contact the potential participant to discuss the study in more detail and if the potential participant is interested a consent visit will be booked. For potential participants at GMMH, preference for an in-person vs. remote consent and baseline appointment will be established at the introductory phone call.  The potential participant will be sent a copy of the Participant Information Sheet and consent form either by email or post, depending upon their preference.  For GMMH potential participants the researcher will document in the participants Health and Social Care record the initial contact. Potential participants will be informed that a contact note will be added to state the date, time and purpose of the call e.g., to introduce the research project. This is in line with GMMH RDSOP35 Use of PARIS in Research. |
| Informed consent and eligibility check | Potential participants will meet with the researcher to discuss the PIS, ask any questions and if they wish to provide informed consent to participate in the study. They will have at least 24 hours to consider the information in the PIS. Informed consent will be taken by a member of the research team to whom this duty has been delegated by the CI (as recorded on the delegation of duties log). The researcher will have training and experience in taking written informed consent.  The consent form and the appointment will be recorded on the participant’s Health and Social Care record at the NHS Trust that is responsible for their care.  COVID-19 Amendments – obtaining informed consent via remote methods  This will involve the researcher taking consent sending a copy of the consent form (either electronically or via the post) to the individual and then asking the individual to provide verbal consent to each item on the consent form. The research worker will make a recording of the verbal consent using an NHS Trust approved encrypted Dictaphone and store this securely on the secure NHS Trust drive. |
| Eligibility check and baseline assessment | Participants will be offered choices regarding length of the assessment, including the option of breaks and multiple occasions. Assessment measures will be clearly prioritised so that the most important will be collected first to avoid missing data. We will have a standard protocol for managing any distress that is associated with the completion of measures, which we have successfully utilised in several trials and has been developed in collaboration with service users; this includes telephone contact within 48 hours of assessments in order to check on participant well-being and participants will be offered the ‘Helpline Numbers’ card.  Participants will receive a token of appreciation for their participation of £20. This will be offered by bank transfer, voucher or cash (subject to covid-19 restrictions).  Eligible participants will be randomised within two working days of confirming eligibility. Participants will be notified of the outcome of randomisation by letter and if requested by a telephone call in addition to a letter.  Contact will be recorded on the participant’s Health and Social Care Record the NHS Trust responsible for their care. |
| End of treatment assessment (2.5 months) and follow-up (6 months). | All participants will receive an assessment with a research assistant at 2 and 6 months.  Participants will be offered choices regarding length of the assessment, including the option of breaks and multiple occasions. Assessment measures will be clearly prioritised so that the most important will be collected first to avoid missing data. We will have a standard protocol for managing any distress that is associated with the completion of measures, which we have successfully utilised in several trials and has been developed in collaboration with service users; this includes telephone contact within 48 hours of assessments in order to check on participant well-being and participants will be offered the ‘Helpline Numbers’ card  Participants will receive a token of appreciation for their participation of £20. This will be offered by bank transfer, voucher or cash (subject to covid-19 restrictions).  On the final visit the participant will be asked if they would like to be informed of the outcome of the research.  Contact will be recorded on the participant’s Health and Social Care Record the NHS Trust responsible for their care. |
| Let’s Talk intervention | For those allocated to receive the intervention, up to 10 weeks of the Let’s Talk programme will be offered by the Peer Support Worker. Plus, up to one booster session will be delivered by their study Peer Support Worker if requested. We will have a standard protocol for managing any distress that we have successfully utilised in several trials and has been developed in collaboration with service users; this includes telephone contact within 48 hours of assessments in order to check on participant well-being and participants will be offered the ‘Helpline Numbers’ card. All PSWs receive weekly clinical supervision and will be able to access support from a clinical psychologist at the Psychosis Research Unit after a session if required. Contact will be recorded on the participant’s Health and Social Care Record the NHS Trust responsible for their care. |
| Qualitative study | Those allocated to receive Let’s Talk will be offered the option of completing a qualitative interview. The qualitative interview will be semi-structured and informed by the topic guide. It will be delivered by a member of the research team who has been delegated this task by the CI. The interview will last between 45 -60 minutes. The interview will be audio-recorded using a GMMH Trust encrypted Dictaphone.  Participants will be offered the option of a follow-up call within 48 hours to discuss any issues that may have arisen for them after the interview. Those who wish to receive a call will be contacted by the researcher at an agreed time. Participants will be offered the ‘Helpline Numbers’ card.  Participants will receive a token of appreciation for their participation of £10.  Contact will be recorded on the participant’s electronic medical records. |

**Assessment of safety: Recording and reporting Adverse or Serious Adverse Events**

Any untoward medical occurrence will be considered an Adverse Event (AE), including occurrences that are not necessarily cause by or related to the study procedures. An adverse event can therefore be any unfavourable and unintended sign, symptom or disease temporally associated with the study procedures, whether or not considered to be related to the study procedures. This may include incidents of self-harm. A serious adverse event will be defined as all deaths, suicide attempts, serious violent incidents, any other life-threatening incident, admissions to a psychiatric hospital, and formal complaints about the study. We shall record all of these as SAEs. In addition to these, in research other than CTIMPs, the Health Research Authority also classes the following as a SAE: results in persistent or significant disability or incapacity; consists of a congenital anomaly or birth defect; or is s otherwise considered medically significant by the investigator and as such we shall record all of these. Decision making regarding expectedness and seriousness shall be delegated to the Chief Investigator (Pyle) and her mentor and supervisor (Morrison) who is a Professor of Clinical Psychology. This may also be delegated to another clinically qualified member of the research team at Pyle & Morrison discretion. This will be recorded on the delegation of duties log. In deciding expectedness GCP Guidance will be referred to, as follows: “Expectedness is determined by comparing the symptoms with the available information related to the study procedures or the IMP. References for all studies are the Protocol and Patient Information Sheet”. When categorising an adverse event as serious and/or unexpected, GCP Guidance will be referred to, as follows: “An SAE occurring to a research participant should be reported to the main REC where in the opinion of the Chief Investigator (CI) the event was: (1) Related – that is, it resulted from administration of any of the research procedures, **and** (2) Unexpected – that is, the type of event is not listed in the protocol as an expected occurrence”.

All adverse events and serious adverse events will be recorded on a standardised AE/SAE form and database, developed from previous NIHR funded clinical trials. Any SAEs deemed to be unexpected and related will be reported to the REC by the CI or another member of the research team delegated with this duty as per the delegation of duties log. This will be done immediately to ensure notification has been sent to the REC within 15 days of the Chief Investigator first being made aware of the event.

NHS Trust incident reporting systems will be in parallel with SAE reporting system at both NHS sites in line with their local policies and procedures. Both sites research related SAEs will be reported to sponsor R&I.

**Risks and anticipated benefits for trial participants**

This study will contribute to the evidence base for the range of interventions to reduce the impact of stigma and discrimination by providing much needed feasibility data. The feasibility data will be used to support an application for further funding to conduct a larger feasibility RCT.

A potential risk is that some participants might find topics discussed distressing. Participants will be reminded at the start and throughout the assessments and intervention that they are able to take breaks or stop at any point. Participants will always be given the choice to decline a question if they wish to do so and researchers and peer support workers are trained to ask questions in a sensitive and non-judgemental. The measures selected for the assessment have been carefully considered and only essential assessment measures have been included to ensure that the assessment battery is minimal. Assessment measures will be clearly prioritised so that the most important will be collected first.

The researchers and peer support workers are trained to respond to any distress that arises. In addition, Dr Elizabeth Murphy and Professor Tony Morrison will be available to provide advice regarding minimising distress. At the Psychosis Research Unit (PRU), which is part of GMMH we have a standardised protocol for managing distress, which has been developed with service users, this includes offering telephone contact within 48 hours of assessments in order to check on participant wellbeing and a summary of helpline numbers.

The number of Peer Support Workers in NHS secondary mental health care settings is growing in the UK and The Mental Health Implementation Plan identifies opportunities for the growth of peer support workers up until 2024 (NHS England, 2019). However, we recognise that some of the topics discuss in peer support about stigma and discrimination may potentially be upsetting. The peer support workers who will provide peer support on the trial are currently employed by GMMH NHS Foundation Trust and are trained to manage any incidences of distress in a sensitive and non-judgemental manner. If any risks to self or others arise the peer support workers have mandatory training in safeguarding vulnerable adults and children, are trained in the Psychosis Research Unit’s safe working policy (including calling and out to a safety checker) and will have direct access to clinical supervision from either Dr Elizabeth Murphy (co-investigator and clinical psychologist at PRU), or Tony Morrison (principle investigator and professor of clinical psychology at PRU). All peer support workers will have Good Clinical Practice Training and understand Information Governance and confidentiality issues.

**Data collection and management**

Each study participant will be assigned a unique study identification number at the start of their involvement with the study. This number will be written on all forms/datasheets, databases and transcripts. No personally identifying information will be written on study forms (other than the consent form that has first and last name) including transcripts. A hard copy of a record sheet linking patient name, medical record ID and study identification number for all participants will be retained as the participant key. It will be the only document that links the anonymised study ID to the participants name and electronic medical record ID number. It will be stored on a password protected Microsoft Excel file, sorted on a secure NHS Drive that is only accessible to the research team and in paper format with consent forms (to facilitate audit of the participant key and consent forms) in a locked NHS cabinet and office. It will be stored separately to any research data.

Referral forms will contain PID. They will be completed electronically in a Microsoft word document and saved password protected on a secure NHS Drive only accessible to the research team. The password will only be shared with the research team. Consent forms will be stored in a locked NHS cabinet and office and separate to any research data. The research register will be stored as a Microsoft excel document. All research data will be pseudo-anonymised with a participant study ID number. Anonymised research data will be entered into the Research Electronic Data Capture (REDCap) system, a secure web platform for building and managing online databases.

With consent some of the assessments, peer support sessions and qualitative interviews will be audio recorded using a GMMH NHS Foundation Trust approved Encrypted Dictaphone. The audio recording will be upload and encrypted as an interview audio file on secure NHS Drive at the earliest possible convenience after the interview. Qualitative interviews will be transcribed verbatim, and transcripts will have all personally identifiable information removed.

All data will be kept secure at all times and maintained in accordance with the requirements of GDPR and archived according to GCP regulations. In line with the sponsor’s Standard Operating Procedures (RDSOP21), all data obtained from NHS patients will be retained for 5 years after the end of the study. Specific retention dates are as follows:

- Research assessments, qualitative interview transcripts, informed consent forms and informed consent audio recordings (where consent was taken remotely) will be retained for 5 years after the end of the study i.e., 2028 in line with the GMMH SOP RDSOP21.
- Audio recordings of peer support sessions, or qualitative interviews will be destroyed after fidelity ratings have been carried out and verified/ after qualitative analysis has taken place.
- The participant key will be retained for 3 years after the study ends and destroyed in 2026.
- Referral forms will be retained for 12 months after the end of the study and destroyed in 2024.

Data will be archived and destroyed in line with the sponsors SOP (RDSOP21).

With written informed consent we will access the electronic health and social care record of participants. Access will be designated to members of the research team by the Chief Investigator (Pyle) for the purpose of: (1) uploading the consent form, (2) entering contact notes (3) checking suitability for home visits/ up-to-date safeguarding information, confirmation of demographic information and a check on adverse/serious adverse events during the period of participation in the study. Members of the research team to whom is delegated will have completed training for the electronic health and social care record, have an up-to-date Information Governance certificate issued by the NHS Trust and have completed International Conference on Harmonisation (ICH) Guidelines - Good Clinical Practice (GCP). Delegation of this duty will be recorded on the delegation of duties log and retained in the site file.

**Statistical analysis plan**

All main analyses will be based on the Intention-to-Treat (ITT) principle. Analysis will take place after full recruitment and follow-up. The main focus will be on tabulated and associated graphical summaries of the key indicators of success of the pilot, including participant recruitment; checks for absence of selective recruitment of participants; baseline balance and participant flow. We will report data in line with the Consolidated Standards of Reporting Trials (CONSORT) 2010 extension statement for Pilot and Feasibility trials showing attrition rates and loss to follow-up. Important summary statistics will be the number of participants referred through mental health staff, number of referrals found to be eligible, and number of consenting individuals and recruited individuals to each arm. Numbers for drop-out from the allocated interventions, withdrawal of consent and failure to provide follow-up outcome data will be generated. We will report our feasibility results (recruitment, retention, adherence) overall, in order to inform decisions about the viability of a future definitive trial. However, we will also report our descriptive results and 95% confidence intervals on outcome measures by group. This will include checking responses and administration methods for the Economic Patient Questionnaire.

**Qualitative Analysis Plan**

Data will be analysed using an inductive Thematic Analysis approach (Braun & Clarke, 2006), with the researcher, taking an interpretative stance to identify stakeholders views of the acceptability and implementation issues for the intervention and necessary adaptations for the UK context. All data will be analysed within the software Nvivo. Each transcript will be coded once transcription is complete. The following steps to thematic analysis will be taken, although, this should not be viewed as a linear model, where one cannot proceed to the next phase without completing the prior phase:

Familiarisation with the data through reading and re-reading the data (and listening to audio-recorded data at least once, if relevant) and noting any initial analytic observations.

Coding by generating pithy labels for important features of the data of relevance to the (broad) research question guiding the analysis capturing both a semantic and conceptual reading of the data. The researcher codes every data item and ends this phase by collating all their codes and relevant data extracts.

Searching for themes: A theme is a coherent and meaningful pattern in the data relevant to the research question. Searching’ is an active process with the researcher constructing themes. The researcher ends this phase by collating all the coded data relevant to each theme.

Reviewing themes: this will involve checking that the themes ‘work’ in relation to both the coded extracts and the full dataset. It may be necessary to collapse two themes together or to split a theme into two or more themes, or to discard the candidate themes altogether and begin again the process of theme development.

Defining and naming themes: Requires the researcher to conduct and write a detailed analysis of each theme.

Writing up to weave together the analytic narrative with data extracts to tell the reader a coherent and persuasive story about the data.

Analysis will be conducted by a member of the research team with experience in qualitative methodology and will be carried out under the supervision of an expert in qualitative research and methodology (Peters).

**Management of the study**

Greater Manchester Mental Health Foundation Trust will be the sponsor. In accordance with high standards of research governance we would ensure researchers receive training in the International Conference on Harmonisation (ICH) Guidelines - Good Clinical Practice before recruitment commences. The study will be managed by the Chief Investigator (Pyle). Pyle has been a co-investigator and the trial manager for multiple NIHR-funded, multi-site RCTs and an open trial of peer support for people with psychosis. Morrison will provide Pyle with weekly mentoring meetings; as assistant director (research) of the Psychosis Research Unit, Pyle receive weekly mentoring to which mentoring for this project will be added through the additional funding resource. Morrison has been chief investigator of multiple multi-centre NIHR-funded RCTS of psychosocial interventions for psychosis. The team have an active research interest in reducing stigma for people with psychosis (Corrigan, Pyle, Wood, Morrison), including having conducted RCTs with self-stigma as primary outcome (Corrigan, Morrison, Wood, Pyle). Jones has expertise in organising and facilitating PPI involvement in research and conducting qualitative research. Peters, who has extensive experience in conducting and supervising qualitative research and will have oversight of phase providing supervision to Jones and Pyle. Corrigan is the originator of a model of self-stigma and the HOP intervention that was developed on this basis.

**End of Study**

Recruitment will end on31^st^ January 2023. Research assessments will end31^st^ July 2023. The study end date is30^th^ August 2023. The end of study is classed as when all the data is analysed. The CI will agree with the sponsor an archiving plan in line with the sponsors SOP RDSOP21.

**Ethics and Regulatory Approvals**

The investigator and sponsor will permit study related monitoring, audits and Health Research Authority (HRA) review and regulatory inspection, providing direct access to source documents and data where required.

The study will not commence until ethical, HRA, sponsor green light and NHS Trust Capacity and Capability reviews and approval has been provided.

**Quality Assurance**

All members of the research team undertaking research activities outlined in this protocol will receive training in the protocol and specific tasks allocated to them i.e., on the listed on the delegation of duties log and the training log.

Research assessment data collection and scoring will be conducted by the Let’s Talk research assistant who will be supervised by Pyle (Chief Investigator) who is an expert in the field of stigma and psychosis with over 35 academic publications in the field, over a decades experience of trial management and the supervision of research assistant, having supervised 28 research assistants. Pyle will monitor completion of consent forms, research assessments and data entry to ensure accuracy of completion and entry. The qualitative interview will be conducted by experienced researchers in the field of psychosis (Jones, Pyle, Peters) and will be conducted with oversight from an expert in qualitative research (Peters). Transcription and analysis will be conducted by an experienced qualitative researcher (Jones) who is an NHS employee, this will be carried out with oversight from an expert in qualitative research (Peters) that will include regular meetings to review thematic structure.

**Dissemination and Publications**

The Chief Investigator (Pyle) will be responsible for writing publications and the publication policy, aspects of this may be delegated to another member of the research team as per the delegation of duties log.

**Finance and Insurance**

This study is funded by the National Institute for Health Research (NIHR) via the Research for Patient Benefit Scheme (RfPB) and is sponsored by Greater Manchester Mental Health NHS Foundation Trust. Insurance and indemnity are provided via the sponsor.

**References**

Addington D, Addington J, Schissel B. (1990). A depression rating scale for schizophrenics. Schizophrenia Research, 3(4), 247-51.

Alonso, J., et al. (2011). Association of Perceived Stigma and Mood and Anxiety Disorders: Results from the World Mental Health Surveys. Acta Psychiatria Scandanavica, 118(4), 305-314.

Angermeyer, M.C.,Matschinger, H. (2003). Public beliefs about schizophrenia and depression: similarities and differences. Soc Psychiatry Psychiatr Epidemiol, 38(9), 526-43.

Birchwood, M., et al. (1993). Depression, demoralization and control over psychotic illness: a comparison of depressed and non-depressed patients with a chronic psychosis. Psychological Medicine, 23, 387-395.

Boyd, J.E., Otilingam, P.G. & DeForge, B.R. (2014). Brief version of the Internalized Stigma of Mental Illness (ISMI) scale: psychometric properties and relationship to depression, self-esteem, recovery orientation, empowerment, and perceived devaluation and discrimination. Psychiatric Rehabilitation Journal, 37(1), pp. 17–23.

Brohan, E., Elgie, R., Sartorius, N., & Thornicroft, G. (2010). Self-stigma, empowerment and perceived discrimination among people with schizophrenia in 14 European countries: The GAMIAN-Europe study. Schizophrenia Research, 122(1), 232-238.

Burke, E., et al. (2018). The effects of peer’s support on empowerment, self-efficacy, and internalized stigma: A narrative synthesis and meta-analysis. Stigma and Health, <http://dx.doi.org/10.1037/sah0000148>

Bobes J, García-Portilla P, Sáiz PA, Bascarán T, Bousoño M. (2005). Quality of life measures in schizophrenia. European Psychiatry, 20(3), S313-S7.

Cook, David R. (1987). Measuring shame: The Internalized Shame Scale. Alcoholism Treatment Quarterly, 4(2), 197-215. doi: 10.1300/J020v04n02_12

Corrigan, P.W., et al. (2010). Self‐stigma and coming out about one's mental illness. Journal of Community Psychology, 38(3), 259-275.

Corrigan PW, Sokol KA, Rusch N. (2013). Reducing self-stigma by coming out proud. American Journal of Public Health. 2013;103(5):794-800.

Davies LM, Barnes TRE, Jones PB, Lewis S, Gaughran F, Hayhurst K, et al. (2008). A Randomized Controlled Trial of the Cost-Utility of Second-Generation Antipsychotics in People with Psychosis and Eligible for Clozapine. Value in Health, 11(4), 549-62.

Gillard, S. , Banach, N., Barlow, E., Byrne, J., Foster, R., Goldsmith, L., Marks, J., McWilliam, C., Morshead, R., Stepanian, K., Turner, R., Verey, A. and White, S. (2021). Developing and testing a principle-based fidelity index for peer support in mental health services. Social Psychiatry and Psychiatric Epidemiology, doi: 10.1007/s00127-021-02038-4

Goffman, E. (1963). Stigma: Notes on the management of a spoiled identity. New York: Simon & Schuster.

Hill, K., Startup, M. (2013). The relationship between internalized stigma, negative symptoms and social functioning in schizophrenia: the mediating role of self-efficacy. Psychiatry Research, 206, 151- 157.

Law H, Neil ST, Dunn G, Morrison AP. (2014). Psychometric properties of the Questionnaire about the Process of Recovery (QPR). Schizophrenia Research, 156(2), 184-9.

Lecomte, T., Corbiere, M., & Laisne, F. (2006). Investigating self-esteem in individuals with schizophrenia: Relevance of the Self-Esteem Rating Scale-Short Form. Psychiatry Research, 143, 99-108.

Mulfinger, N., et al. (2018). Honest, Open, Proud for adolescents with mental illness: pilot randomised controlled trial. Journal of Child Psychology and Psychiatry, 59 (6), 684-91.

National Institute for Health and Care Excellence, Psychosis and schizophrenia in adults: treatment and management. Clinical Guideline 178. 2014, UK: NICE

Oexle, N., et al. (2017). Mental illness stigma, secrecy and suicidal ideation. Epidemiologia a Psichiatria Sociale, 26 (1), 53-60.

Pontin E, Peters S, Lobban F, Rogers A, Morriss RK. (2009). Enhanced relapse prevention for bipolar disorder: a qualitative investigation of value perceived for service users and care coordinators. Implementation Science, 4:4-.

Priebe S, Huxley P, Knight S, Evans S. (1999). Application and results of the Manchester Short Assessment of Quality of Life (MANSA). International Journal of Social Psychiatry, 45, 7-12.

Peters S, Pontin E, Lobban F, Morriss R. (2011). Involving relatives in relapse prevention for bipolar disorder: a multi-perspective qualitative study of value and barriers. BMC Psychiatry, 11:172-.

Rogers, E.S., et al. (1997). A consumer-constructed scale to measure empowerment among users of mental health services. Psychiatric Services, 48(8), 1042-1047.

Rusch, N., et al. (2009a). A stress-coping model of mental illness: II. Emotional stress response, coping behavior and outcome. Schizophrenia Research, 110 (1-3), 65-71.

Rusch, N., et al. (2009b). A stress-coping model of mental illness stigma: I. Predictors of cognitive stress appraisal. Schizophrenia Research, 110(1-3), 59-64.

Rusch, N., et al. (2014). Efficacy of Coming Out Proud to reduce stigma's impact among people with mental illness: a pilot randomised controlled trial. British Journal of Psychiatry, 204, 391-397.

Kösters, M. & Rüsch, N. (2021). Honest, Open, Proud to support disclosure decisions and to decrease stigma’s impact among people with mental illness: conceptual review and meta‑analysis of program efficacy. Social Psychiatry and Psychiatric Epidemiology, <https://doi.org/10.1007/s00127-021-02076-y>

Sibitz, I., et al., The impact of the social network, stigma and empowerment on the quality of life in patients with schizophrenia. European Psychiatry, 2011. 26(1): p. 28-33.

Taskforce, M.H., The Five Year Forward View for Mental Health: A report from the independent Mental Health Taskforce to the NHS in England, 2016, NHS England: England.

Upthegrove, R., et al. (2017). Depression and Schizophrenia: Cause, Consequence, or Trans-diagnostic Issue? Schizophrenia Bulletin, 2017. 43(2): p. 240 - 244.

Vass, V., et al. (2015). How stigma impacts on people with psychosis: The mediating effect of self-esteem and hopelesness on subjective recovery and psychotic experiences. Psychiatry Research, 15(2), 487-95.

Vauth R, Kleim B, Wirtz M, Corrigan P. (2007). Self-efficacy and empowerment as outcomes of self-stigmatizing and coping schizophrenia. Psychiatry Research, 150:71 – 80

Watson, A.C., et al. (2007). Self-stigma in people with mental illness. Schizophrenia Bulletin, 33, 1312 – 1318

Whitehead, A.L., Julious, S.A., Cooper, C.L., Campbell, M.J. (2016). Estimating the sample size for a pilot randomised trial to minimise the overall trial sample size for the external pilot and main trial for a continuous outcome variable. Statistical Methods in Medical Research, 25(3) 1057 –1073.

Wood, L., et al. (2016). Semi-structured Interview Measure of Stigma (SIMS) in psychosis: Assessment of psychometric properties. Schizophrenia Research, 176(2), 398-403.

Yi Chong, H., et al. (2016). Global economic burden of schizophrenia: a systematic review. Neuropsychiatry of Disease and Treatment, 12, 357-373.

# **Intervention details**

**Let’s Talk Workbook Sections**

| **Section #** | **Details** |
| --- | --- |
| Section 1 | Getting to know each other: (1) expectations of the intervention, (2) what is peer support, (3) getting to know each other’s hobbies/ interests, values and important relationships, (4) getting to know each other in regard to mental health and psychosis experiences, and preferred terms for mental health experiences. |
| Section 2 | Talking about mental health: (1) negative and positive terms about mental health and psychosis, (2) hurtful (stigmatising) and helpful words about psychosis, (3) finding out facts to challenge stigma including myths psychosis vs. facts about psychosis with links to recovery stories and normalising information in the workbook appendix. |
| Section 3 | Hurtful and helpful self-talk about mental health experiences: (1) sharing hurtful and helpful self-talk understanding & experiences, (2) analysing a story for hurtful and helpful self-talk, and (3) five steps for changing personally hurtful self-talk. |
| Section 4 | Disclosure options: (1) reasons people choose to disclose, (2) benefits and costs of disclosure, (3) short-term and long-term costs and benefits of disclosure, (4) Five points to remember about disclosure decisions, and (5) my benefits and costs analysis. |
| Section 5 | Choices and settings for disclosure: (1) six approaches to talking about mental health, (2) benefits and costs of the six approaches including examples and own benefit and cost analysis, (3) Who is a good person to disclose to? Evaluating types of disclosure relationships and testing the water for a good person to disclose to. |
| Section 6 | How others may react to your disclosure: (1) evaluating helpful and unhelpful reactions, (2) evaluating possible experiences after an unhelpful reaction including an option to role play an unhelpful reaction, and (3) ways to prepare or manage an unhelpful reaction. |
| Section 7 | Sharing information about your mental health experience: (1) different ways to share your mental health experiences, (2) Creating your own approach to sharing your experiences of mental health and psychosis, (3) a structured guide to setting up your own narrative about your mental health and psychosis experiences, and (4) practicing sharing your experiences. |
| Section 8 | Moving forward: (1) Evaluating your experience of disclosure, (2) insights about the intervention and future directions and (3) where to go to find further peer support and connection. |
| Appendix | Appendix 1: National and local organisations that can provide mental health support.  Appendix 2: Example costs and benefits of disclosing.  Appendix 3: Example costs and benefits of disclosure by social media.  Appendix 4: Examples of characteristics and qualities of people and relationships for talking about mental health.  Appendix 5: Recovery stories and resources about psychosis/  Appendix 6: Local peer support groups.  Appendix 7: signposting regarding protection again unwanted disclosures and legal rights regarding disclosure.  Appendix 8: Disclosure narrative example. |

**Peer Principles for Let’s Talk delivery**

Let’s Talk is operating in a statutory setting where the values of peer support may be experienced differently to voluntary or community settings. Peer Support Workers (PSWs) will be in paid employment in NHS settings with mandatory responsibilities such as note keeping and reporting risk. However, as a peer-delivered approach our baseline intention is to deliver Let’s Talk within a principle’s framework.

For this reason, a set of operational principles developed by Gillard et al. (2017) will be used to inform Let’s Talk. These principles were chosen because they were derived from through systematic review of research about one-to-one peer support and a review of a set of principles produced by a UK National Expert Panel of people sharing, leading or researching peer support from a lived experience perspective. The research team that produced the peer support principles below included researchers working from a lived experience perspective.

Principles based approach to Peer Support developed by Gillard et al.

**Principle 1: Support the building of safe, trusting relationships based on shared lived experience.** For example:

1. Providing people with a safe, non-judgemental environment in which the supported peer can share their experiences of stigma, mental health, and psychosis.
2. The PSW will be supported to respond appropriately and safely when people share difficult or emotional life experiences through training, individual and group supervision with a peer specialist and clinical psychologist.
3. Ensuring that the PSWs feel safe to share their own experiences.

**Principle 2: Ensure that the values of mutuality and reciprocity underpin the peer relationship. For example:**

1. The PSW and supported peer will share their experiences across stigma, disclosure, and psychosis dimensions.
2. PSW and peers will understand the person’s experience from their lived experience perspective.
3. The sessions for Let’s Talk will foster a feeling of solidarity with each other.
4. Both PSW and supported peer can learn from each other.

**Principle 3: Promote the validation and application of experiential knowledge in the delivery of peer support. For example:**

1. Helping people learn from their experience and incorporate it and/or move forward.

**Principle 4: Enable peers to exercise leadership, choice and control over the way in which peer support is given and received. For example:**

1. PSW will promote the supported peer making a choice in the length, frequency, and settings for Let’s Talk sessions.
2. PSW will promote choice in how exercises are completed or choosing to miss an exercise.
3. The Let’s Talk PSW will be supported to make choice and leadership in sharing their own narrative
4. The PSW and supported peer will work together to identify what works best for the supported peer in regards to accessing additional resources on stigma, normalising information and personal narratives that are shared in session or reviewed as a between session task.
5. Supported peers and PSW will be empowering to provide feedback about sessions.

**Principle 5: Empower peers to discover and make use of their own strengths and build and strengthen connections to their peers and wider communities. For example:**

1. In Let’s Talk intervention sessions the PSW will focusing on a person’s strengths, helping them develop their ability to make use of the resources available to them.
2. Where relevant to the supported peer, the Let’s Talk PSW will utilise their knowledge and experience of peer communities to foster connections for the supported peer with the wider peer community.

# **Fidelity Scale**

**Overview of Fidelity for the Let’s Talk Programme**

Treatment fidelity is the extent to which an intervention is accurately implement as it was designed i.e. how accurately it reflects the purpose (values/principles) and content of an intervention (Wilczynski et al., 2017).

For the Let’s Talk programme we will evaluate how accurately a session reflects the workbook content and the extent to which it was deliverable using the principles of peer support.

The competency in delivery of workbook content will be evaluating using the Let’s Talk Programme Fidelity Scale that utilises a scoring system of 0 (absence of any discussion relating to the content) – 6 (very good features with minimal problems/ inconsistencies with the workbook content). This scale is adapted from the Dreyfus Model of Competence incorporated in the Revised Version of the Cognitive Therapy Rating Scale (CTS-R), this scale evaluates the ability of the person delivering the workbook content. The Let’s Talk workbook is divided into material for eight sessions lasting approximately one hour each.

The principles of peer support will be evaluated using an adapted version of the Principles Based Fidelity Index (Gillard et al., 2021).

Throughout the lifetime of the trial a random selection of audio recorded session will be selected to evaluate fidelity using both the Let’s Talk Programme Fidelity Scale and the Principles Based Fidelity Index. The Let’s Talk team have collaboratively agreed that fidelity ratings will be completed as a group, forming part of group supervision. The group supervision sessions are attended by peer support workers, a peer specialist, the Chief investigator and a clinical psychologist. In addition, we will supplement the Let’s Talk Programme Fidelity Scale and the Principles Based Index with data from individual session records and end of treatment records completed by the Peer Support Worker.

**Let’s Talk Programme Fidelity Scale**

**Instructions**

Three areas will be assessed for each session rated:

1. General ability in delivery
2. Workbook section ability
3. Actual vs. planned content covered in the session

Using the 0-6 rating scale, each session should first be rated on the four general items. A score and a written rationale to support the score should be provided.

The rater should then list each workbook section covered in the session and score using the workbook section specific scoring criteria provided below. Whilst it is expected that sessions will be linear in the delivery i.e. from sessions 1-8, it is possible that a supported peer and peer support worker may choose to start a session by finishing off a previous session, peers may choose to revisit previous sessions to review information/ add new information. Rating the workbook specific sections covered within the rated session will allow for both planned or unplanned non-linear progression and provide data on the actual vs. planned workbook sections covered. For each workbook section covered in the session the rater should use the 0-6 rating scale to provide a score and written rationale to support the score. Each session is likely to contain between 3-5 workbook sections for review. A score sheet for the Let’s Talk Fidelity rating score is in Appendix 1.

**Rating Scale for general and workbook sections**

0 - Absence of any discussion of the workbook section outlined for the session

1 - Major problem evident with delivering the workbook section outlined for the session

2 - Evidence of ability, but several problems in delivering the workbook section outlined for the session and lack of consistency

3 – Able in the delivery with some minor problems in delivering the workbook section outlined for the session

4 - Good features, but minor problems in delivering the workbook section outlined for the session

5 - Very good features, minimal problems and/or inconsistencies in delivering the workbook section outlined for the session

6 - Excellent, covering all aspects of the workbook section outlined for the session

Please note that the top marks of 6 are **reserved** for highly effective ability, particularly in the face of difficulties. We set a minimum competence standard of a score of 3 marks per item.

**General items**

| **Item** | **Considerations for the rater** |
| --- | --- |
| Session rationale and reference to the material | The PSW:   - Provides the session overview/ objectives are discussed in line with the manual description - References the workbook material throughout the session in a manner that accords with the content e.g. directing to appropriate pages for the workbook sections, directing to relevant appendix content, directing to appropriate tables/ information provision |
| Flexibility and Choice | The PSW:   - Offers choice in how workbook material is completed for that session providing a flexible approach. - Supports and facilitates the supported peer’s choices regarding delivery for the session. |
| Sharing | The PSW:   - Where comfortable and appropriate the peer support worker discloses something about themselves that is relevant to the topic area being covered for the session. Shared interests outside of the session content or mental health topic do not generally count for fidelity scores except for session 1 (building the relationship). - Where sharing occurs it provides one of the following functions: role modelling (relevant to the topics in the workbook), it increases connectedness/ sense of being alone (e.g. I was in a similar situation once), provides a different perspective on a problem or conveys a message of hope. |
| Feedback | The PSW:   - Invites the supported peer to feedback their opinion of the previous session and discusses their responses accordingly. - Invites the supported peer to feedback on the current session and discusses responses accordingly. - Shares at least one positive feature of the session/ strength of the peer that they will take away/ have identified |

**Workbook specific sections**

| **Workbook session #** | **Workbook section** | **Considerations for the rater** |
| --- | --- | --- |
| 1 | Expectations and questions about the Let’s Talk programme | The PSW:   - Elicits discussion with the supported peer about their expectations of the programme. - Invites the supported peer to ask questions about the programme. - Responds to the supported peer’s expectations and questions. |
|  | What is a Peer? | The PSW:   - Explains that a peer is someone who shares a similar life experience. - Provides an example for the supported peer of how they share a similar lived experience regarding mental health. - Emphasises working together, or alongside each other. |
|  | Getting to know each other | The PSW:   - Elicits information about the supported peer’s interests - Shares information with the supported peer about their own interests/identity - Discuss the supported peers preferred mental health terms. - Shares with the supported peer their preferred terms they used regarding their mental health. |
| 2 | Negative and Positive Attitudes Towards Mental Health | The PSW:   - Facilitates conversation about the key features of stigma (stereotypes, prejudice, and discrimination) and how this can relate to disclosure decisions. - Facilitates conversation regarding examples of positive attitudes towards mental health and how this can relate to disclosure decisions. - Invites the peer to add/ supplement information in workbook with their own examples and discusses with the peer their responses. |
|  | Hurtful and helpful words and actions | The PSW:   - Facilitates conversation regarding helpful/ hurtful words the supported peer is aware of regarding mental health and the personal impact on disclosure decisions. - Invites the supported peer to discuss aspects of identity, other than mental health, where they have experienced/ are concerned about experiencing stigma. - If the peer identifies intersections with other forms of discrimination the PSW actively listens, validates, recognises the peer’s perspectives on the intersection of stigma and offers signposting support. |
|  | Finding out facts to challenge stigma | The PSW:   - Outlines the position of the Let’s Talk programme regarding stereotypes and prejudice as false and unfair. - Outlines how information provision including recovery stories can address stigma/ myths. - Offers the supported peer options for information provision and explores what would be most relevant for the supported peer. - Shares at least one example of information provision within the workbook appendix/ leaflets that they have found helpful. |
| 3 | Helpful and hurtful self-talk | The PSW:   - Shares how hurtful self-talk, because of stigma, is sometimes referred to as internalised stigma and this is an understandable reaction to hearing or experiencing negative attitudes from others. - Elicits feedback on the examples of hurtful self-talk - Shares the definition of helpful self-talk - Elicits feedback on the examples of helpful self-talk |
|  | Analysing a story for Hurtful & Helpful Self-Talk/ Recognising hurtful and helpful self-talk | The PSW:   - Facilitates the supported peer to identify helpful and hurtful self-talk in other people’s stories using Amy and Pete’s examples provided in the workbook. - Facilitates conversation with the supported peer regarding the supported peer’s own helpful and hurtful self-talk. - Validates and encourages the supported peers helpful self-talk, or if the supported peer is unable to identify any helpful self-talk the PSW offers to share strengths that they have identified in the peer. - Highlights the information provided in Appendix 5 may help with hurtful self-beliefs because of stigma |
|  | 5 Step-approach | The PSW:   - Describes the 5-step approach as an optional self-help tool to challenge hurtful self-beliefs. - Shares information about the 5-step approach with the supported peer, outlining each of the five steps. - Elicits feedback on the 5-step approach from the support peer - Confirms if the supported peer would like to complete it. - If the supported peer chooses to complete the 5-step approach: The PSW draws on their own experience of completing the 5-step approach to help guide the peer through the exercise if the peer chooses to complete it. |
| 4 | Reasons some people choose to talk about their mental health | The PSW:   - Shares with the supported peer the reasons why others choose to talk about their mental health and elicits feedback. - Identifies if the supported peer has additional reasons they would add to the list and discusses. - Shares any additional reasons that they would add to the list. |
|  | Benefits and costs of talk about mental health | The PSW :   - Shares the workbook definition of benefits and costs and invites feedback. - Supports the supported peer in identifying their own benefits and costs to talking about their mental health and NOT talking about their mental health. - Supports the supported peer to identify their own personal benefits and costs of not talking about their mental health problems. - Shares the concept of short- and long-term benefits and cost and elicits discussion with the supported peer regarding the case example. |
|  | Five points to remember about disclosure decisions | The PSW:   - Shares each of the five points regarding disclosure decisions outlined in the workbook and elicits feedback from the supported peer. - Invites the peer to consider if disclosure via social media platforms is relevant for them - If social media platforms are relevant for the supported peer: The PSW supports, the peer to consider the benefits and costs using Appendix 3. |
|  | My benefits and cost analysis | The PSW:   - Supports the supported peer in identifying a setting and a person for the benefits and cost analysis. - Elicits the supported peer’s perspective on the short-term and long-term benefits. - Elicits the supported peer’s to identify which of the short and long-term benefits and costs are most important to them. - Supports the peer in identify a goal for disclosure. - Supports the peer to consider possible reactions to a disclosure in the specified setting and with the specified person are considered. - Supports the peer to use the benefit and cost analysis to practice making a disclosure decision. |
| 5 | Six approaches to talking about mental health | The PSW:   - Shares with the supported peer the six approaches to talking about mental health including example benefits and costs. - Elicits the supported peer’s perspectives regarding the six approaches to talking about mental health. - Supports the supported peer to consider their own benefits and costs to each approach. |
|  | Who is a Good Person to Disclose to? | The PSW:   - Highlights the link between a disclosure goal and the type of person/ relationships for a disclosure. - Supports the supported peer to consider three types of relationships (supportive, functional, and empathic) and elicits the peer’s perspective regarding the qualities of a person who fall into each relationship category. - Shares the concept of ‘testing the water’. - Elicits a response from the supported peer regarding their appraisal of the five potential responses to a conversation about mental health. - The PSW supports the peer to evaluate responses to a conversation about mental health. - Supports the supported peer to design their own testing the water exercise. |
| 6 | Helpful and unhelpful reactions | The PSW:   - Facilitates conversation regarding helpful and unhelpful emotional reactions to a disclosure and the supported peer is invited to add their own examples to the possible reactions list. - Offers the supported peer the option to complete the ‘*experiences after an unhelpful reaction’* exercise - If the supported peer chooses to do the ‘experiences after an unhelpful reaction’ exercise they are supported to choose how they wish to complete this exercise e.g. decide on their own example reaction, completing as a conversation vs. role play. - If the supported peer chooses to complete the ‘experiences after an unhelpful reaction, they are invited to complete the three rating scales and the interpretation of the ratings discussed. - If the peer chooses to complete the ‘experience after an unhelpful reaction’ exercise facilitates conversation regarding how the supported peer they felt pre, during and after the role play/ whether they found it helpful or not. - If the peer chooses to do the ‘experience after an unhelpful reaction exercise the debrief is provided. |
|  | Ways to prepare for and manage hurtful or unhelpful reactions | The PSW:   - Shares the strategies listed to prepare for or manage an unhelpful reaction to a disclosure and elicits a response from the supported peer. - Invites the supported peer to suggest other ways to manage an unhelpful reaction. - Shares information in Appendix 7 on protection against unwanted disclosures. - Supports the supported peer to create their own individualised plan for taking care of their wellbeing during a time where they have decided to disclose/ are considering disclosure. |
| 7 | Ways to share your mental health experiences | The PSW:   - Facilitates conversation regarding the various ways listed to sharing information about mental health experiences. - Highlights the links to examples of the various ways to sharing information about mental health including those listed in the appendix. - Draws links between and the ways listed to share experiences and approaches to disclosure discussed in session five e.g. broadcast or selective. |
|  | Creating your own approach to sharing your mental health experiences | The PSW:   - Facilitates conversation regarding the four guiding questions i.e. who, what, how much. - Supports the supported peer to identify their own preference/s regarding sharing their mental health experiences. - Draws on the supported peers examples of goals for disclosure, settings, and sharing preferences discussed from this and previous sessions. - Outlines an approach they have taken to sharing information about their mental health experiences. |
|  | A guide to sharing your mental health experiences | The PSW:   - Highlights that each topic area is optional and that the purpose is not to collect data on the peer. - Facilitates the conversation, eliciting feedback regarding each section that the peer chooses to complete. - Invites the supported peer to review the guide and cross through information they would choose not to share and circling information they consider important. - Invite to practice sharing their information from the guide with PSW, or plan to share outside of the session if the supported peer wishes (options should be provided including sharing with another Let’s Talk PSW; research team member; peer group; or another person the supported peer chooses). If outside the session the supported peer should be guided through a plan. |
| 8 | Evaluating the peers experience of a disclosure | The PSW:   - Supports the supported peer to complete an evaluation of their experience using three main reflection points: the goal of disclosing, the summary of what was said, the persons reactions. - Actively listens and supports discussion regarding the peer’s experience of a disclosure. - Invites the supported peer to complete the two rating scales for evaluating an experience of disclosure, highlighting the peer should not discount any feelings. - Discusses the peers responses using the rating scale provided in the workbook. |
|  | Insights and future directions | The PSW:   - Supports the supported peer to evaluate their experience of completing Let’s Talk across eight areas using the questions provided, discussing their responses - Shares their own learning from working alongside the peer for the Let’s Talk programme. - Shares with the peer information about peer services from Appendix six and creates a master list of peer support groups relevant for the peer. - Offers and where relevant plans the booster session with the peer. |

Principles Based Fidelity Index

| **V1. During the course of all the peer support sessions…** | **Never** | **Sometimes** | **Always** | **NA** |
| --- | --- | --- | --- | --- |
| 1. The supported peer and PSW agreed how they would work together |  |  |  |  |
| Comments: | | | |  |
| 2. The supported peer and PSW talked openly and constructively about things that were difficult and challenging |  |  |  |  |
| Comments: | | | |  |
| 3. It was possible for the PSW to meaningfully share their lived experience with the supported peer. |  |  |  |  |
| Comments: | | | |  |
| 4. The PSW utilised their own judgement to support the supported peer regarding risk and safety |  |  |  |  |
| Comments: | | | |  |
| 5. The PSW properly discussed and agreed anything that they might share about the supported peer with another mental health worker |  |  |  |  |
| Comments: | | | |  |
| 6. The PSW took take time to understand how the supported peer made sense of their mental health experiences |  |  |  |  |
| Comments: | | | |  |
| 7. There was evidence of mutual respect between the PSW and the supported peer |  |  |  |  |
| Comments: | | | |  |
| 8. The PSW learnt something new from the supported peer |  |  |  |  |
| Comments: | | | |  |
| 9. The PSW and supported peer agreed what, if anything, was to be recorded in notes made about the session(s) |  |  |  |  |
| Comments: | | | |  |
| 10. A two-way sharing and listening process was established between the PSW and the supported peer |  |  |  |  |
| Comments: | | | |  |
| 11. The supported peer was encouraged to be in control of what I they shared with the PSW |  |  |  |  |
| Comments: | | | |  |
| 12. It was possible to allow the supported peer to decide what happened when they met with the PSW, rather than being directed by the PSW |  |  |  |  |
| Comments: | | | |  |
| 13. The PSW encouraged the supported peer to think about and do the things they enjoy doing |  |  |  |  |
| Comments: | | | |  |
| 14. The PSW encouraged the supported peer to think about and get in touch with the people they like spending time with |  |  |  |  |
| Comments: | | | |  |
| 15. The PSW encouraged the supported peer to think about and go to places they enjoy visiting |  |  |  |  |
| Comments: | | | |  |
| 16. The PSW encouraged the supported peer to think about the future (once we stop working together) |  |  |  |  |
| Comments: | | | |  |

PSW = Peer Support Worker; Score 0 for never/ disagree; 1 for sometimes/ partly agree; 2 for always/ fully agree

Total score in the range 0-30

|  | |
| --- | --- |
| **Name of rater** |  |
| **Participant ID** |  |
| **Session No.** |  |
| **Exercises and talking points covered in the session** |  |
| **General items** | |
| Session rationale and reference to the material | Score:  Rationale: |
| Flexibility and choice | Score:  Rational: |
| Sharing | Score:  Rationale: |
| Feedback | Score:  Rationale: |
| **General items total score:** | |
| **Session specific content** | |
| Workbook section covered:  Score:  Rationale | |
| Workbook section covered  Score:  Rationale: | |
| Workbook section covered  Score:  Rationale: | |
| Workbook section covered:  Score:  Rationale: | |
| Workbook section covered:  Score:  Rationale: | |
| Workbook section covered:  Score:  Rationale | |
| Workbook section covered:  Score:  Rationale | |

# **Results**

**Table 1: Participant exclusions**

|  | **Reason**  **n (%)** |
| --- | --- |
| **Declined at referral** | **N=25** |
| No reason | 10 (40.0) |
| Too busy | 8 (32.0) |
| Wrong time | 4 (16.0) |
| On different project | 3 (12.0) |
| **Ineligible at referral** | **N=9** |
| No stigma or distress concerns | 4 (44.4) |
| No F20-F29 diagnosis and not under EIP | 2 (22.2) |
| Moved out of area | 1 (11.1) |
| Require interpreter | 1 (11.1) |
| No care team | 1 (11.1) |
| **Uncontactable post referral** | **N=27** |

Values are either numbers or numbers (percent). EIP Early Intervention in Psychosis; F20-F29 schizophrenia spectrum diagnosis.

**Table 2: Reasons for change of status**

|  | **Let’s Talk + TAU N=35** | **TAU N=35** |
| --- | --- | --- |
| Number of participants who withdrew consent to participate | **n=3 (8.6)** | **n=2 (5.7)** |
| **Reason** |  |  |
| Did not want to continue | 1 (33.3) | 1 (50.0) |
| No reason given | - | 1 (50.0) |
| Reported that they found some of the content of the workbook upsetting specifically the line in Pete's story "I wasn’t so sure I wanted to be around either" and that talking about past experiences was difficult | 1 (33.3) | - |
| Participant reported that they are not in a good place mentally and feels unable to continue with sessions as they ‘are not in the correct mindset at present | 1 (33.3) | - |

Values are numbers (percent). TAU treatment as usual

**Table 3: Referral pathway**

|  | **Referred N=149** | **Excluded at referral N=61** | **Provided informed consent N=88** | **Excluded after informed consent N=18** | **Randomised N=70** | **Randomised to Let’s Talk + TAU N=35** | **Randomised to TAU N=35** |
| --- | --- | --- | --- | --- | --- | --- | --- |
| **Location and service** |  |  |  |  |  |  |  |
| Greater Manchester Mental Health Foundation Trust | | | |  |  |  |  |
| EIP | 91 (61.1) | 40 (65.6) | 51 (58.0) | 10 (55.6) | 41 (58.6) | 20 (57.1) | 21 (60.0) |
| CMHT | 39 (26.2) | 13 (21.3) | 26 (29.5) | 7 (38.9) | 19 (27.1) | 10 (28.6) | 9 (25.7) |
| Northeast London Foundation Trust | |  |  |  |  |  |  |
| CMHT/CRT | 19 (12.8) | 8 (13.1) | 11 (12.5) | 1 (5.6) | 10 (14.3) | 5 (14.3) | 5 (14.3) |

Values are n (percent). EIP Early Intervention in Psychosis; CMHT Community Mental Health Team; CRT Community Recovery Team.

**Table 4 : Reasons for receiving less than half the sessions available.**

|  | **N =12** |
| --- | --- |
| Multiple non-attendance or cancellation of sessions within the intervention window limiting number of sessions available to deliver the intervention. | 7 (58.3) |
| Withdrew from trial | 2 (16.7) |
| Hospitalised for period of intervention | 1 (8.3) |
| Declined intervention (no reason provided) | 1 (8.3) |
| Declined: not the right time to do the intervention due to challenge life situation | 1 (8.3) |

**Table 5 Secondary outcomes**

|  | **Let’s Talk + TAU N=35** | **TAU N=35** | **MD** | **95% CI** |
| --- | --- | --- | --- | --- |
| **The Semi-structed Interview Measure for Stigma in Psychosis** |  |  |  |  |
| Total^1^ |  |  |  |  |
| Baseline | 23.2 (5.7); 35 | 22.2 (4.1); 35 |  |  |
| 2.5 Months | 15.5 (6.4); 31 | 18.6 (5.2); 26 | -3.31 | (-6.03, -0.59) |
| 6 Months | 12.8 (6.5); 27 | 17.3 (6.0); 28 | -4.83 | (-7.57, -2.08) |
| Internalised stigma total^1^ |  |  |  |  |
| Baseline | 18.5 (4.5); 35 | 18.1 (3.8); 35 |  |  |
| 2.5 Months | 12.6 (5.9); 31 | 15.3 (4.7); 26 | -2.57 | (-5.06, -0.08) |
| 6 Months | 10.0 (5.9); 27 | 14.3 (5.5); 28 | -4.19 | (-6.71, -1.68) |
| Perceived stigma total^1^ |  |  |  |  |
| Baseline | 2.7 (0.8); 35 | 2.6 (0.7); 35 |  |  |
| 2.5 Months | 2.2 (0.7); 31 | 2.4 (0.8); 26 | -0.31 | (-0.67, 0.04) |
| 6 Months | 2.1 (1.0); 27 | 2.2 (0.6); 28 | -0.19 | (-0.55, 0.16) |
| Experienced stigma total^1^ |  |  |  |  |
| Baseline | 2.0 (1.2); 35 | 1.6 (1.1); 35 |  |  |
| 2.5 Months | 0.7 (0.8); 31 | 0.8 (0.7); 26 | -0.26 | (-0.66, 0.13) |
| 6 Months | 0.7 (0.9); 27 | 0.8 (0.9); 28 | -0.27 | (-0.67, 0.14) |
| **Stigma Stress Scale** |  |  |  |  |
| Difference score ^1^ |  |  |  |  |
| Baseline | 0.6 (7.7); 28 | 2.5 (7.6); 30 |  |  |
| 2.5 Months | -1.7 (9.7); 29 | 1.4 (7.7); 23 | -2.33 | (-6.65, 1.99) |
| 6 Months | -5.0 (8.2); 24 | -1.7 (8.7); 25 | -2.37 | (-6.77, 2.04) |
| Perceived harm^1^ |  |  |  |  |
| Baseline | 20.5 (5.2); 29 | 21.4 (5.1); 30 |  |  |
| 2.5 Months | 18.7 (6.5); 29 | 21.4 (5.7); 24 | -1.71 | (-4.44, 1.02) |
| 6 Months | 17.7 (6.7); 25 | 19.5 (5.6); 28 | -1.02 | (-3.74, 1.71) |
| Perceived resources^2^ |  |  |  |  |
| Baseline | 20.3 (4.7); 28 | 18.9 (5.7); 30 |  |  |
| 2.5 Months | 20.4 (4.8); 29 | 19.7 (4.2); 23 | 0.40 | (-2.11, 2.91) |
| 6 Months | 22.2 (4.2); 24 | 20.6 (5.5); 25 | 1.34 | (-1.21, 3.89) |
| **The process of recovery questionnaire^2^** |  |  |  |  |
| Baseline | 29.9 (14.6); 27 | 31.5 (12.0); 29 |  |  |
| 2.5 Months | 35.9 (10.9); 24 | 30.6 (14.7); 19 | 4.04 | (-1.78, 9.86) |
| 6 Months | 36.9 (11.7); 21 | 34.6 (13.9); 26 | 3.61 | (-2.11, 9.33) |
| **Disclosure distress scale^1^** |  |  |  |  |
| Baseline | 5.8 (1.1); 35 | 5.8 (1.2); 35 |  |  |
| 2.5 Months | 4.2 (1.8); 31 | 5.3 (1.4); 25 | -0.94 | (-1.77, -0.11) |
| 6 Months | 4.2 (1.8); 27 | 4.9 (1.8); 27 | -0.65 | (-1.49, 0.19) |
| **The Calgary depression scale^1^** |  |  |  |  |
| Baseline | 9.9 (4.9); 29 | 11.1 (6.0); 29 |  |  |
| 2.5 Months | 7.8 (5.3); 26 | 9.8 (6.0); 22 | -1.10 | (-3.62, 1.43) |
| 6 Months | 6.6 (4.5); 20 | 9.2 (6.3); 26 | -1.33 | (-3.89, 1.24) |
| **EQ-5D-5L^2^** |  |  |  |  |
| Baseline | 0.455 (0.380); 18 | 0.511 (0.339); 21 |  |  |
| 2.5 Months | 0.514 (0.334); 16 | 0.502 (0.260); 15 | -0.023 | (-0.191, -0.191) |
| 6 Months | 0.478 (0.310); 11 | 0.484 (0.362); 14 | 0.022 | (-0.155, -0.155) |
| **Social Interaction Anxiety Scale^1^** |  |  |  |  |
| Baseline | 37.8 (22.9); 21 | 40.1 (15.2); 25 |  |  |
| 2.5 Months | 27.1 (14.5); 12 | 43.5 (15.9); 13 | -10.45 | (-20.78, -0.12) |
| 6 Months | 36.1 (16.2); 12 | 38.6 (19.9); 12 | -5.04 | (-15.39, 5.32) |
| **Rogers Empowerment Scale^1^** |  |  |  |  |
| Total |  |  |  |  |
| Baseline | 95.3 (13.9); 20 | 92.9 (11.1); 18 |  |  |
| 2.5 Months | 90.8 (8.7); 17 | 93.9 (11.7); 17 | -2.00 | (-7.62, 3.61) |
| 6 Months | 93.2 (9.5); 13 | 90.3 (10.9); 10 | 2.71 | (-3.57, 9.00) |
| Optimism and Control Over Future |  |  |  |  |
| Baseline | 10.2 (3.0); 21 | 9.6 (2.4); 20 |  |  |
| 2.5 Months | 9.6 (2.3); 18 | 9.7 (2.5); 19 | -0.36 | (-1.75, 1.03) |
| 6 Months | 10.6 (1.9); 13 | 9.1 (2.7); 14 | 0.91 | (-0.66, 2.49) |
| Self-esteem and self-efficacy |  |  |  |  |
| Baseline | 23.2 (6.5); 21 | 22.7 (6.9); 19 |  |  |
| 2.5 Months | 20.1 (4.3); 17 | 22.3 (6.3); 17 | -0.56 | (-3.43, 2.30) |
| 6 Months | 22.9 (6.3); 13 | 20.7 (5.9); 14 | 2.86 | (-0.09, 5.81) |
| Power |  |  |  |  |
| Baseline | 44.2 (3.1); 20 | 43.8 (3.0); 20 |  |  |
| 2.5 Months | 43.0 (4.0); 17 | 44.1 (3.0); 19 | -1.02 | (-2.99, 0.95) |
| 6 Months | 42.8 (3.2); 13 | 42.8 (3.7); 12 | 0.03 | (-2.27, 2.32) |
| Community activism and autonomy |  |  |  |  |
| Baseline | 9.0 (3.3); 21 | 8.8 (2.2); 19 |  |  |
| 2.5 Months | 9.6 (2.4); 18 | 9.5 (3.2); 19 | 0.18 | (-1.24, 1.61) |
| 6 Months | 9.2 (2.6); 13 | 8.6 (1.7); 14 | 0.43 | (-1.22, 2.08) |
| Righteous anger |  |  |  |  |
| Baseline | 19.4 (2.3); 21 | 20.1 (2.5); 20 |  |  |
| 2.5 Months | 19.5 (2.3); 19 | 19.9 (2.5); 19 | -0.00 | (-1.49, 1.49) |
| 6 Months | 18.4 (3.4); 13 | 19.1 (1.9); 12 | -0.61 | (-2.38, 1.16) |
| **Self-esteem rating scale^2^** |  |  |  |  |
| Total |  |  |  |  |
| Baseline | 76.8 (26.2); 17 | 76.1 (24.4); 17 |  |  |
| 2.5 Months | 81.1 (26.2); 17 | 75.0 (27.3); 15 | -0.44 | (-15.05, 14.18) |
| 6 Months | 82.5 (24.2); 11 | 81.8 (32.9); 15 | -0.86 | (-15.96, 14.24) |
| Positive |  |  |  |  |
| Baseline | 38.6 (16.4); 17 | 38.6 (11.8); 18 |  |  |
| 2.5 Months | 40.9 (14.5); 17 | 36.7 (12.7); 15 | 0.81 | (-6.40, 8.02) |
| 6 Months | 41.9 (12.7); 11 | 39.2 (15.3); 15 | 1.98 | (-5.67, 9.64) |
| Negative |  |  |  |  |
| Baseline | 43.4 (13.5); 18 | 42.1 (13.7); 17 |  |  |
| 2.5 Months | 40.1 (15.2); 18 | 41.7 (15.8); 15 | 0.78 | (-7.29, 8.85) |
| 6 Months | 39.5 (12.3); 11 | 37.4 (18.2); 15 | 3.06 | (-5.27, 11.40) |
| **Internalised Shame Scale^1^** |  |  |  |  |
| Total shame |  |  |  |  |
| Baseline | 88.3 (20.5); 18 | 83.0 (25.7); 16 |  |  |
| 2.5 Months | 74.5 (23.9); 17 | 75.1 (22.8); 16 | -6.24 | (-18.76, 6.28) |
| 6 Months | 76.7 (22.8); 11 | 74.6 (30.6); 14 | 0.39 | (-12.80, 13.58) |
| Total self esteem |  |  |  |  |
| Baseline | 16.9 (7.2); 18 | 16.2 (5.5); 17 |  |  |
| 2.5 Months | 18.4 (6.4); 18 | 17.9 (6.1); 16 | -0.75 | (-4.02, 2.51) |
| 6 Months | 18.6 (5.6); 11 | 17.9 (6.3); 14 | -0.54 | (-4.05, 2.97) |
| **Manchester Short Assessment^2^** |  |  |  |  |
| Baseline | 49.2 (16.2); 20 | 52.2 (11.2); 23 |  |  |
| 2.5 Months | 58.7 (10.8); 19 | 49.8 (8.3); 15 | 5.64 | (-1.63, 12.92) |
| 6 Months | 59.4 (11.5); 13 | 54.3 (16.1); 16 | 4.87 | (-2.76, 12.49) |

Values are mean (standard deviation); n.

^1^ A lower score indicates a better outcome for this measure/ subscale.

^2^ A higher score indicates a better outcome for this measure/ subscale.

**Table 6: Peer Fidelity Index across all sessions**

|  | **Disagree**  **N=182** | **Partly agree N=182** | **Fully agree N=182** | **Missing N=182** |
| --- | --- | --- | --- | --- |
| The supported peer and I agreed how we would work together | 1 (0.5) | 7 (3.8) | 172 (94.5) | 2 (1.1) |
| The supported peer and I were able to talk openly and constructively about things that were difficult and challenging | 1 (0 .5) | 10 (5.5) | 163 (89.6) | 8 (4.4) |
| It was possible to meaningfully share my lived experience with the supported peer | 2 (1.1) | 29 (15.9) | 143 (78.6) | 8 (4.4) |
| I felt confident using my own judgement to support the supported peer regarding risk and safety | 32 (17.6) | 4 (2.2) | 138 (75.8) | 8 (4.4) |
| I was able to properly discuss and agree anything that I might share about the supported peer with another mental health worker | 58 (31.9) | 33 (18.1) | 83 (45.6) | 8 (4.4) |
| I was able to take time to understand how the supported peer made sense of their mental health experiences | 3 (1.6) | 4 (2.2) | 167 (91.8) | 8 (4.4) |
| I feel that there was mutual respect between the supported peer and I | 2 (1.1) | 7 (3.8) | 165 (90.7) | 8 (4.4) |
| I learned something new from the supported peer | 1 (0.5) | 10 (5.5) | 163 (89.6) | 8 (4.4) |
| The supported peer and I agreed what, if anything, was to be recorded in notes made about the session(s) | 35 (19.2) | 40 (22.0) | 99 (54.4) | 8 (4.4) |
| A two-way sharing and listening process was established between the supported peer and I | 3 (1.6) | 10 (5.5) | 161 (88.5) | 8 (4.4) |
| The supported peer was encouraged to be in control of what I they shared with me | 1 (0.5) | 16 (8.8) | 157 (86.3) | 8 (4.4) |
| It was possible to allow the supported peer to decide what happened when we met, rather than being directed by myself as the peer support worker | 1 (0.5) | 75 (41.2) | 98 (53.8) | 8 (4.4) |
| I encouraged the supported peer to think about and do the things they enjoy doing | 69 (37.9) | 13 (7.1) | 91 (50.0) | 9 (4.9) |
| I encouraged the supported peer to think about and get in touch with the people they like spending time with | 82 (45.1) | 46 (25.3) | 46 (25.3) | 8 (4.4) |
| I encouraged the supported peer to think about and go to places they enjoy visiting | 91 (50.0) | 46 (25.3) | 37 (20.3) | 8 (4.4) |
| I encouraged the supported peer to think about the future (once we stop working together) | 92 (50.5) | 27 (14.8) | 56 (30.8) | 7 (3.8) |

Values are n (%).

**Table 7: Hospital use at Baseline**

|  | **Let’s Talk + TAU N=35** | **TAU N=35** |
| --- | --- | --- |
| Planned hospital overnight stays |  |  |
| No | 16 (45.7) | 18 (51.4) |
| Yes | 3 (8.6) | 3 (8.6) |
| Missing | 16 (45.7) | 14 (40.0) |
| Attended hospital outpatient appointments for less than 4 hours |  |  |
| No | 14 (40.0) | 13 (37.1) |
| Yes | 5 (14.3) | 8 (22.9) |
| Missing | 16 (45.7) | 14 (40.0) |
| Attended hospital outpatient appointments for more than 4 hours (but not overnight) |  |  |
| No | 19 (54.3) | 19 (54.3) |
| Yes | - | 2 (5.7) |
| Missing | 16 (45.7) | 14 (40.0) |
| Attended accident and emergency (A&E) services |  |  |
| No | 13 (37.1) | 13 (37.1) |
| Yes | 6 (17.1) | 8 (22.9) |
| Missing | 16 (45.7) | 14 (40.0) |
| Attended any primary and community based health services |  |  |
| No | 1 (2.9) | - |
| Yes | 18 (51.4) | 20 (57.1) |
| Don't know | - | 1 (2.9) |
| Missing | 16 (45.7) | 14 (40.0) |
| GP (surgery) |  |  |
| No | 4 (11.4) | 6 (17.1) |
| Yes | 15 (42.9) | 13 (37.1) |
| Don't know | - | 2 (5.7) |
| Missing | 16 (45.7) | 14 (40.0) |
| GP (home) |  |  |
| No | 18 (51.4) | 18 (51.4) |
| Yes | 1 (2.9) | 2 (5.7) |
| Don't know | - | 1 (2.9) |
| Missing | 16 (45.7) | 14 (40.0) |
| Practice Nurse (surgery) |  |  |
| No | 8 (22.9) | 11 (31.4) |
| Yes | 11 (31.4) | 8 (22.9) |
| Don't know | - | 2 (5.7) |
| Missing | 16 (45.7) | 14 (40.0) |
| Nurse (home) |  |  |
| No | 18 (51.4) | 15 (42.9) |
| Yes | 1 (2.9) | 5 (14.3) |
| Don't know | - | 1 (2.9) |
| Missing | 16 (45.7) | 14 (40.0) |
| Other physical care service (walk in centre) |  |  |
| No | 13 (37.1) | 13 (37.1) |
| Yes | 6 (17.1) | 7 (20.0) |
| Don't know | - | 1 (2.9) |
| Missing | 16 (45.7) | 14 (40.0) |
| Other physical care service (other) |  |  |
| No | 19 (54.3) | 12 (34.3) |
| Yes | - | 7 (20.0) |
| Don't know | - | 1 (2.9) |
| Missing | 16 (45.7) | 15 (42.9) |
| Mental Health Services (counsellor/mental health worker) |  |  |
| No | 3 (8.6) | - |
| Yes | 16 (45.7) | 20 (57.1) |
| Don't know | - | 1 (2.9) |
| Missing | 16 (45.7) | 14 (40.0) |
| Other social support services (social worker) |  |  |
| No | 16 (45.7) | 17 (48.6) |
| Yes | 2 (5.7) | 1 (2.9) |
| Don't know | - | 1 (2.9) |
| Missing | 17 (48.6) | 16 (45.7) |
| Other social support services (home help) |  |  |
| No | 18 (51.4) | 18 (51.4) |
| Don't know | - | 1 (2.9) |
| Missing | 17 (48.6) | 16 (45.7) |
| Other social support services (care worker) |  |  |
| No | 12 (34.3) | 11 (31.4) |
| Yes | 6 (17.1) | 8 (22.9) |
| Don't know | - | 1 (2.9) |
| Missing | 17 (48.6) | 15 (42.9) |
| Other social support services (occupational therapist) |  |  |
| No | 18 (51.4) | 18 (51.4) |
| Don't know | - | 1 (2.9) |
| Missing | 17 (48.6) | 16 (45.7) |
| Other social support services (physiotherapist) |  |  |
| No | 18 (51.4) | 16 (45.7) |
| Yes | - | 2 (5.7) |
| Don't know | - | 1 (2.9) |
| Missing | 17 (48.6) | 16 (45.7) |
| Other social support services (other) |  |  |
| No | 12 (34.3) | 12 (34.3) |
| Yes | 6 (17.1) | 7 (20.0) |
| Don't know | - | 1 (2.9) |
| Missing | 17 (48.6) | 15 (42.9) |
| Other health or social care or support services last 12 months |  |  |
| No | 12 (34.3) | 11 (31.4) |
| Yes | 7 (20.0) | 6 (17.1) |
| Don't know | - | 3 (8.6) |
| Missing | 16 (45.7) | 15 (42.9) |

Values are n (%)

**Table 8: Hospital use at 2.5 months follow-up**

|  | **Let’s Talk + TAU N=35** | **TAU N=35** |
| --- | --- | --- |
| Planned hospital overnight stays |  |  |
| No | 14 (40.0) | 15 (42.9) |
| Yes | 2 (5.7) | 1 (2.9) |
| Missing | 19 (54.3) | 19 (54.3) |
| Attended hospital outpatient appointments for less than 4 hours |  |  |
| No | 9 (25.7) | 14 (40.0) |
| Yes | 7 (20.0) | 2 (5.7) |
| Missing | 19 (54.3) | 19 (54.3) |
| Attended hospital outpatient appointments for more than 4 hours (but not overnight) |  |  |
| No | 14 (40.0) | 15 (42.9) |
| Yes | 2 (5.7) | 1 (2.9) |
| Missing | 19 (54.3) | 19 (54.3) |
| Attended accident and emergency (A&E) services |  |  |
| No | 14 (40.0) | 13 (37.1) |
| Yes | 2 (5.7) | 3 (8.6) |
| Missing | 19 (54.3) | 19 (54.3) |
| Attended any primary and community based health services |  |  |
| No | 1 (2.9) | 2 (5.7) |
| Yes | 15 (42.9) | 14 (40.0) |
| Missing | 19 (54.3) | 19 (54.3) |
| GP (surgery) |  |  |
| No | 9 (25.7) | 9 (25.7) |
| Yes | 7 (20.0) | 6 (17.1) |
| Missing | 19 (54.3) | 20 (57.1) |
| GP (home) |  |  |
| No | 15 (42.9) | 13 (37.1) |
| Yes | 1 (2.9) | 2 (5.7) |
| Missing | 19 (54.3) | 20 (57.1) |
| Practice Nurse (surgery) |  |  |
| No | 14 (40.0) | 13 (37.1) |
| Yes | 2 (5.7) | 2 (5.7) |
| Missing | 19 (54.3) | 20 (57.1) |
| Nurse (home) |  |  |
| No | 16 (45.7) | 13 (37.1) |
| Yes | - | 2 (5.7) |
| Missing | 19 (54.3) | 20 (57.1) |
| Other physical care service (walk in centre) |  |  |
| No | 15 (42.9) | 14 (40.0) |
| Yes | - | 1 (2.9) |
| Don't know | 1 (2.9) | - |
| Missing | 19 (54.3) | 20 (57.1) |
| Other phsyical care service (other) |  |  |
| No | 14 (40.0) | 10 (28.6) |
| Yes | 2 (5.7) | 4 (11.4) |
| Don't know | - | 1 (2.9) |
| Missing | 19 (54.3) | 20 (57.1) |
| Mental Health Services (counsellor/mental health worker) |  |  |
| No | 3 (8.6) | 3 (8.6) |
| Yes | 13 (37.1) | 12 (34.3) |
| Missing | 19 (54.3) | 20 (57.1) |
| Other social support services (social worker) |  |  |
| No | 15 (42.9) | 13 (37.1) |
| Yes | 1 (2.9) | 1 (2.9) |
| Missing | 19 (54.3) | 21 (60.0) |
| Other social support services (home help) |  |  |
| No | 16 (45.7) | 14 (40.0) |
| Missing | 19 (54.3) | 21 (60.0) |
| Other social support services (care worker) |  |  |
| No | 14 (40.0) | 10 (28.6) |
| Yes | 2 (5.7) | 5 (14.3) |
| Missing | 19 (54.3) | 20 (57.1) |
| Other social support services (occupational therapist) |  |  |
| No | 16 (45.7) | 13 (37.1) |
| Yes | - | 1 (2.9) |
| Missing | 19 (54.3) | 21 (60.0) |
| Other social support services (physiotherapist) |  |  |
| No | 16 (45.7) | 12 (34.3) |
| Yes | - | 2 (5.7) |
| Missing | 19 (54.3) | 21 (60.0) |
| Other social support services (other) |  |  |
| No | 6 (17.1) | 10 (28.6) |
| Yes | 10 (28.6) | 4 (11.4) |
| Missing | 19 (54.3) | 21 (60.0) |
| Other health or social care or support services last 12 months |  |  |
| No | 10 (28.6) | 10 (28.6) |
| Yes | 6 (17.1) | 6 (17.1) |
| Missing | 19 (54.3) | 19 (54.3) |

Values are n (%)

**Table 9: Hospital use at 6 months follow-up**

|  | **Let’s Talk + TAU N=35** | **TAU N=35** |
| --- | --- | --- |
| Planned hospital overnight stays |  |  |
| No | 10 (28.6) | 15 (42.9) |
| Yes | 1 (2.9) | - |
| Missing | 24 (68.6) | 20 (57.1) |
| Attended hospital outpatient appointments for less than 4 hours |  |  |
| No | 8 (22.9) | 12 (34.3) |
| Yes | 3 (8.6) | 3 (8.6) |
| Missing | 24 (68.6) | 20 (57.1) |
| Attended hospital outpatient appointments for more than 4 hours (but not overnight) |  |  |
| No | 10 (28.6) | 15 (42.9) |
| Don't know | 1 (2.9) | - |
| Missing | 24 (68.6) | 20 (57.1) |
| Attended accident and emergency (A& E) services |  |  |
| No | 8 (22.9) | 13 (37.1) |
| Yes | 3 (8.6) | 2 (5.7) |
| Missing | 24 (68.6) | 20 (57.1) |
| Attended any primary and community based health services |  |  |
| No | - | 2 (5.7) |
| Yes | 11 (31.4) | 10 (28.6) |
| Don't know | - | 3 (8.6) |
| Missing | 24 (68.6) | 20 (57.1) |
| GP (surgery) |  |  |
| No | 5 (14.3) | 7 (20.0) |
| Yes | 6 (17.1) | 5 (14.3) |
| Don't know | - | 1 (2.9) |
| Missing | 24 (68.6) | 22 (62.9) |
| GP (home) |  |  |
| No | 11 (31.4) | 11 (31.4) |
| Yes | - | 1 (2.9) |
| Don't know | - | 1 (2.9) |
| Missing | 24 (68.6) | 22 (62.9) |
| Practice Nurse (surgery) |  |  |
| No | 8 (22.9) | 6 (17.1) |
| Yes | 3 (8.6) | 6 (17.1) |
| Don't know | - | 1 (2.9) |
| Missing | 24 (68.6) | 22 (62.9) |
| Nurse (home) |  |  |
| No | 11 (31.4) | 12 (34.3) |
| Don't know | - | 1 (2.9) |
| Missing | 24 (68.6) | 22 (62.9) |
| Other phsyical care service (walk in centre) |  |  |
| No | 11 (31.4) | 11 (31.4) |
| Yes | - | 1 (2.9) |
| Don't know | - | 1 (2.9) |
| Missing | 24 (68.6) | 22 (62.9) |
| Other phsyical care service (other) |  |  |
| No | 9 (25.7) | 10 (28.6) |
| Yes | 2 (5.7) | 2 (5.7) |
| Don't know | - | 1 (2.9) |
| Missing | 24 (68.6) | 22 (62.9) |
| Mental Health Services (counsellor/mental health worker) |  |  |
| No | 1 (2.9) | 1 (2.9) |
| Yes | 10 (28.6) | 11 (31.4) |
| Don't know | - | 1 (2.9) |
| Missing | 24 (68.6) | 22 (62.9) |
| Other social support services (social worker) |  |  |
| No | 11 (31.4) | 12 (34.3) |
| Don't know | - | 1 (2.9) |
| Missing | 24 (68.6) | 22 (62.9) |
| Other social support services (home help) |  |  |
| No | 11 (31.4) | 12 (34.3) |
| Don't know | - | 1 (2.9) |
| Missing | 24 (68.6) | 22 (62.9) |
| Other social support services (care worker) |  |  |
| No | 7 (20.0) | 8 (22.9) |
| Yes | 4 (11.4) | 4 (11.4) |
| Don't know | - | 1 (2.9) |
| Missing | 24 (68.6) | 22 (62.9) |
| Other social support services (occupational therapist) |  |  |
| No | 11 (31.4) | 12 (34.3) |
| Don't know | - | 1 (2.9) |
| Missing | 24 (68.6) | 22 (62.9) |
| Other social support services (physiotherapist) |  |  |
| No | 11 (31.4) | 12 (34.3) |
| Don't know | - | 1 (2.9) |
| Missing | 24 (68.6) | 22 (62.9) |
| Other social support services (other) |  |  |
| No | 6 (17.1) | 6 (17.1) |
| Yes | 5 (14.3) | 6 (17.1) |
| Don't know | - | 1 (2.9) |
| Missing | 24 (68.6) | 22 (62.9) |
| Other health or social care or support services last 12 months |  |  |
| No | 9 (25.7) | 10 (28.6) |
| Yes | 2 (5.7) | 4 (11.4) |
| Don't know | - | 1 (2.9) |
| Missing | 24 (68.6) | 20 (57.1) |

Values are n (%)

**Table 10: Serious and adverse events by allocation**

|  | **Let’s Talk + TAU N=35** | **TAU N=35** |
| --- | --- | --- |
| Number of participants with an SAE/AE | 8 (22.9) | 5 (14.3) |
| Number of SAE/AE | 15 | 13 |
| **Serious adverse events** |  |  |
| Number of participants with an SAE | 6 (17.1) | 3 (8.6) |
| Number of SAE’s | 9 | 6 |
| **Details** |  |  |
| Admission to a psychiatric hospital (voluntary) | 2 | 2 |
| Potentially life-threatening self-harm | 1 | 0 |
| Suicide Attempt | 1 | 3 |
| Admission to a psychiatric hospital (involuntary) | 2 | - |
| Serious violent incident (participant as victim of incident) | 2 | - |
| Serious violent incident | 1 | - |
| Death^1^ | - | 1 |
| **Adverse events** |  |  |
| Number of participants with an AE | 4 (11.4) | 4 (11.4) |
| Number of AE | 6 | 5 |
| **Details** |  |  |
| Increase in suicidal ideation | 1 | 3 |
| Increase in suicidal ideation and behaviours | 2 | - |
| Increase in suicidal ideation requiring intervention | 1 | - |
| Self-harm | - | 1 |
| Other | 2 | 1 |

Values are either n (percent) or n. ^1^ Physical health condition.
